# Augmented Hill-Climb increases reinforcement learning efficiency for language-based de novo molecule generation

**DOI:** 10.1186/s13321-022-00646-z

**Published:** 2022-10-03

**Authors:** Morgan Thomas, Noel M. O’Boyle, Andreas Bender, Chris de Graaf

**Affiliations:** 1 Centre for Molecular Informatics, Department of Chemistry, University of Cambridge, Cambridge, CB2 1EW UK; 2 Computational Chemistry, Sosei Heptares, Steinmetz Building, Granta Park, Great Abington, Cambridge, CB21 6DG UK

**Keywords:** Artificial intelligence, AI, Structure-based drug design, SBDD, Deep learning, Generative models, Recurrent neural network, Molecular docking, Reinforcement learning, De novo design, REINFORCE, Hill-Climb, REINVENT

## Abstract

A plethora of AI-based techniques now exists to conduct de novo molecule generation that can devise molecules conditioned towards a particular endpoint in the context of drug design. One popular approach is using reinforcement learning to update a recurrent neural network or language-based de novo molecule generator. However, reinforcement learning can be inefficient, sometimes requiring up to 10^5^ molecules to be sampled to optimize more complex objectives, which poses a limitation when using computationally expensive scoring functions like docking or computer-aided synthesis planning models. In this work, we propose a reinforcement learning strategy called Augmented Hill-Climb based on a simple, hypothesis-driven hybrid between REINVENT and Hill-Climb that improves sample-efficiency by addressing the limitations of both currently used strategies. We compare its ability to optimize several docking tasks with REINVENT and benchmark this strategy against other commonly used reinforcement learning strategies including REINFORCE, REINVENT (version 1 and 2), Hill-Climb and best agent reminder. We find that optimization ability is improved ~ 1.5-fold and sample-efficiency is improved ~ 45-fold compared to REINVENT while still delivering appealing chemistry as output. Diversity filters were used, and their parameters were tuned to overcome observed failure modes that take advantage of certain diversity filter configurations. We find that Augmented Hill-Climb outperforms the other reinforcement learning strategies used on six tasks, especially in the early stages of training or for more difficult objectives. Lastly, we show improved performance not only on recurrent neural networks but also on a reinforcement learning stabilized transformer architecture. Overall, we show that Augmented Hill-Climb improves sample-efficiency for language-based de novo molecule generation conditioning via reinforcement learning, compared to the current state-of-the-art. This makes more computationally expensive scoring functions, such as docking, more accessible on a relevant timescale.

**Supplementary Information:**

The online version contains supplementary material available at 10.1186/s13321-022-00646-z.

## Introduction

Many generative model techniques and architectures applied to de novo molecule generation exist. These models range from purely symbolic approaches such as genetic algorithms [1, 2] to more recent machine learning (ML) approaches such as recurrent neural networks (RNNs) [3–8], transformers [9–11], variational autoencoders [12–15], generative adversarial networks [16–18], graph neural networks [19, 20] and hybrid approaches that use ML to guide reinforcement learning (RL) in a heuristic action space [21]. These generative models can produce valid and novel molecules [22, 23] and condition molecule generation towards a particular endpoint [22] (e.g., predicted bioactivity towards a protein target [4]) via optimization techniques such as, RL [4, 21, 24], Bayesian optimization [12, 14], molecular swarm optimization [15] and Monte Carlo tree search [2, 6]. Although generative models still face many challenges for trusted and routine integration into drug discovery pipelines including practical relevance and more comprehensive evaluation [25].

Of the more recent ML-based approaches to de novo molecule generation, RNNs were one of the first to appear with the seminal approaches being published about 5 years ago. One study which received wide interest was by Segler et al*.* [3] who fine-tuned an RNN on molecules of biological interest to generate molecules containing similar properties de novo. Another study, by Olivecrona et al*.* [4] instead used RL to update the RNN to generate molecules de novo that maximized predicted properties (e.g., predicted bioactivity of molecules). These results were obtained by representing molecules using the SMILES language [26] which emulates the RNN’s designed application for use in natural language processing [27, 28]. When trained on a large dataset of SMILES (> 10^5^), an RNN can predict the next symbol in a sequence conditional upon previously seen symbols. Thus, by supplying a start symbol, new symbols can be sampled from the probability distribution corresponding to the next symbol (output by the RNN), which is then recursively fed back into the network resulting in de novo molecules. Despite a wave of newer approaches since (e.g., JT-VAE [13], DrugEx [29], GENTRL [30], GraphINVENT [20, 31]), RNNs are still frequently used and investigated for de novo molecule generation (e.g., [32–34]). Furthermore, they still match the state-of-the-art on several de novo molecule generation benchmarks [22, 23, 35, 36].

Although it is possible to optimize RNN de novo molecule generation via fine-tuning on a smaller dataset of molecules relevant to a particular endpoint (as in [3]), such a priori knowledge is not always available or when available, must be used carefully as to not bias de novo molecule generation too much (e.g., resulting in a lack of novelty [37] or very close similarity to fine-tuning datasets which must be monitored [38]). Reinforcement learning (RL) on the other hand can be used to optimize de novo compounds to maximize/minimize a numerical reward which can be provided by either a single or a combination of scoring functions, and it is therefore limited by the accuracy and reliability of scoring functions used and their relevance to the respective objective [39, 40]. Several RL strategies have been combined with RNNs including Hill-Climb (HC) [22, 41], REINFORCE [42] (used in [5]) and REINVENT [4]. Two of these RL strategies (REINVENT and HC) have been shown to rank top one or two in optimization tasks compared to other generative models [22, 35, 36]. Monte Carlo tree search approaches have also been proposed to search a trained RNN’s sample space [6, 43, 44]; however, no RNN parameters are updated (no RNN learning takes place) during this process and so task optimization is rather an optimized search within the RNNs current generative domain.

Despite excellent performance on benchmarks, RNN de novo molecule optimization using RL can be very sample-inefficient often requiring 10 s or 100 s of thousands of molecules to optimize a task. For example, 163,840 molecules were sampled during HC optimization for GuacaMol benchmark tasks [22] and 192,000 molecules were sampled during REINVENT optimization of DRD2 predicted activity [4] (although neither study specified at which point the task was ‘sufficiently’ optimized, which could have been before optimization finished). While low sample-efficiency is not a problem for easily computed scoring functions such as property calculation, it significantly hinders the use of scoring functions requiring a significant amount of computation such as molecular docking and computer aided-synthesis planning. This is becoming increasingly important with recent growth in interest in using molecular docking scoring functions to guide de novo molecule generation [45–53]. This approach has shown to result in more diverse and novel compounds with a broader coverage of known active space than an equivalent QSAR model trained on known ligands [52]. Other studies have used ML to model molecular docking or other physics-based scoring functions which is less computationally expensive [35, 54, 55]. However, use of a model of a model reduces the advantages of such scoring functions by being less able to extrapolate novel chemical space and adds prediction uncertainty on top of pre-existing inaccuracies [56, 57]. Therefore, it is attractive to improve the sample-efficiency of RL optimization to enable routine use of such docking-based scoring functions directly.

Previous work has explored RL strategies and parameters for RNNs de novo molecule generation to varying degrees. Niel et al. [41] compared different RL strategies (including REINFORCE, HC and REINVENT) and optimized a selection of tasks. However, the difference in sample-efficiency was not clear and their code was not published. A comparison of REINVENT versions 1.0 and 2.0 shows that the default sigma parameter value was increased. This effectively increases the reward contribution compared to the prior contribution and theoretically improves sample-efficiency, although this was not discussed in the publication [58]. Fialková et al. [59] investigated more significant modifications to the REINVENT loss function which did not result in any significant improvement. Meanwhile, Atance et al*.* [20] modified the loss function by adding a best agent reminder (BAR) mechanism to the loss function resulting in ‘significantly improved learning’ (although this was not further quantified by the authors and it pertained to use on a graph-based generation model).

Here, with the aim to improve the sample-efficiency of SMILES-based RNNs, we make a very simple change to the REINVENT strategy to ameliorate overpowered regularization by introducing elements of the HC strategy. We call this novel hybrid approach Augmented Hill-Climb (AHC) and investigate it’s use for RNN de novo molecule generation. We further compare AHC to previously mentioned RL strategies that are implemented in published studies and make the code freely accessible [60].

## Methods

**Fig. 1:**
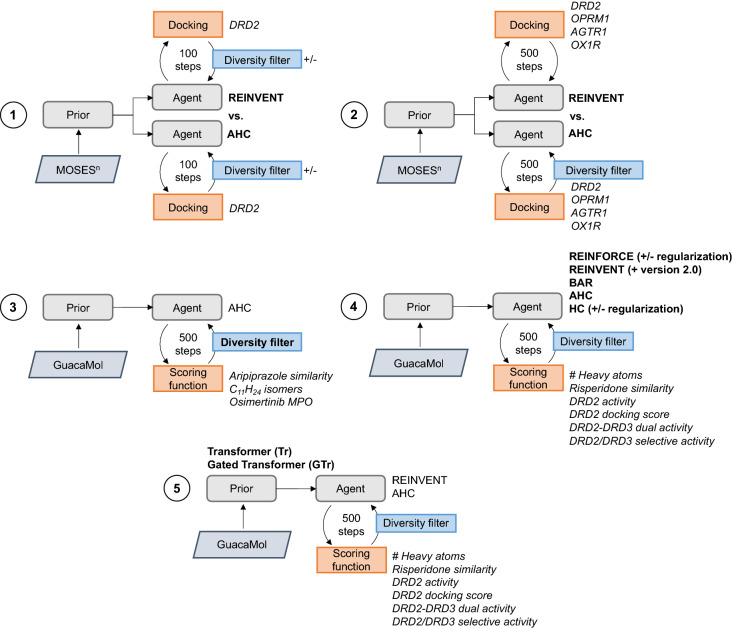
Schematic of the five experiments conducted in this work with the focus of each experiment in bold face. In each case the Prior and Agent refer to an RNN. (1) Comparison of AHC to REINVENT on a single docking task over 100 RL updates. (2) Comparison of AHC to REINVENT on four different docking tasks over 500 RL updates. (3) Diversity filter and parameter search for use in combination with AHC on three toy tasks proposed by GuacaMol benchmark suite. (4) Benchmark comparison of AHC to other RL strategies across a six optimization tasks of varying difficulty. (5) Comparison of AHC to REINVENT on two different transformer architectures on six benchmark tasks

The evaluation of AHC and comparison to other RL strategies was built around five key experiments which are summarised in Fig. 1 (the details of which follow in the remainder of the Methods): Experiment 1, comparison between AHC and REINVENT on the ability to minimize the docking score against the D_2_ receptor (DRD2) over a very limited number of RL updates. Experiment 2, comparison between AHC and REINVENT on the ability to minimize the docking score against four different receptors over an extended number of RL updates relative to Experiment 1. Experiment 3, investigation of diversity filters and their parameters for use in combination with AHC by optimizing toy tasks proposed by the GuacaMol benchmark suite [22]. Experiment 4, benchmark comparison between AHC and other RL strategies on six tasks of varying difficulty. Experiment 5, benchmark comparison between AHC and REINVENT on alternative language-based generative models (a transformer architecture and reinforcement learning stabilized transformer architecture) on the same benchmark tasks as Experiment 4.

### Training data

RNNs were trained using either a modification of the MOSES dataset or the GuacaMol dataset. Firstly, the MOSES dataset [23] is derived from ZINC15 clean leads [61] and contains a library of ‘drug-like’ small organic molecules. It is designed to benchmark generative model de novo molecule generation. The MOSES dataset applies several filters during curation including: molecular weight between 250 and 350 Da; number of rotatable bonds not greater than 8; XlogP [62] not greater than 3.5; no atoms besides C, N, S, O, F, Cl, Br, H; no cycles larger than 7 members; molecules adhering to custom medicinal chemistry [63, 64] and PAINS filters [65]. In addition, charged species are removed; here however, we deviate from this curation by neutralising charged species and hence avoid a bias towards non-protonatable groups. To distinguish this from the original MOSES dataset, we refer to this as MOSES neutralized (MOSES^n^) [52]. This resulted in a training set of 2,454,087 molecules. The GuacaMol train dataset [22] (1,273,104 molecules) is derived from ChEMBL24 and contains real molecules both in the ‘drug-like’ domain and others such as peptides and natural products. This dataset was designed to benchmark both generative model de novo molecule generation and subsequent objective optimization. The GuacaMol dataset applies the following filters during curation: salt removal; charge neutralization; molecules with SMILES strings shorter than 100 characters; no atoms besides H, B, C, N, O, F, Si, P, S, Cl, Se, Br, and I. Therefore, the GuacaMol dataset results in a training set with a much broader variety of chemotypes than MOSES^n^.

### Recurrent neural network

Recurrent neural networks used in this work are deep neural networks composed of layers of either long short-term memory units or gated recurrent units, which store and transfer information from one state to the next. In de novo molecule generation, SMILES symbols are one-hot encoded into a binary vector which is used as input to the network. These networks are then trained to predict the conditional probability of a SMILES subsequent symbol given a sequence of previously seen SMILES symbols. This is achieved by training the network using maximum likelihood estimation (equivalent to minimizing the negative log likelihood), whereby the model must maximize the likelihood assigned to the correct symbol $\mathbf{x}$ at time $\mathbf{t}$ conditional upon all previously observed symbols. The resulting loss function **L** parameterized by the network parameters **θ** is shown in Eq. 1. For further details we refer the reader to [4].

1 $$L\left(\theta \right)= -\sum_{t=0}^{T}logP({x}_{t}|{x}_{t-1}\dots {x}_{0})$$

The RNN implemented in this work is the same as [3, 4, 58, 66]. Specifically, three RNN configurations were used, either trained on MOSES^n^ or GuacaMol train. The first RNN configuration consisted of an embedding layer of size 128 and three gated recurrent unit (GRU) layers of size 512 with no dropout—implemented using the code shared in the original work [4]. This implementation was only used with the original REINVENT RL strategy in experiment 2, as a comparison to older work. The second configuration consisted of an embedding layer of size 256 and three long short-term memory (LSTM) layers of size 512 with no dropout—consistent with the REINVENT 2.0 implementation [58]. The third configuration consisted of three LSTM layers of size 512 with a dropout rate of 0.2, consistent with the GuacaMol implementation [22] as found on the corresponding GitHub repository [67]. The first and second configurations were trained on the MOSES^n^ dataset for 5 epochs using a batch size of 128 with an ADAM optimizer and learning rate of 0.001, while the third configuration was trained on GuacaMol train for 10 epochs using a batch size of 512 with an ADAM optimizer and learning rate of 0.001.

### Transformer

Two transformer encoder architectures were used in this work. The first is the original proposed transformer (Tr) encoder [68]. The second is a gated transformer (GTrXL) encoder adapted from [69] in an attempt to stabilize the transformer under RL conditions. The encoder layer of this second architecture relocates layer normalization before the respective sub-layer (i.e., multi-head attention or feed-forward network) and employs a GRU style gating mechanism in place of the residual connection. Notably the original adaption was applied to Transformer-XL [70] which contains a memory mechanism to expand context for larger language tasks. This memory mechanism was omitted in the current work for simplicity and due to the shorter nature of SMILES strings which are typically 20–100 characters long compared to 10^3^–10^4^ in large language tasks. Therefore, we simply refer to this model as gated transformer (GTr). Lastly, both encoder architectures had a final feed-forward layer to predict the probability over symbols in the vocabulary. In both cases, character masking was used to train the encoder in an autoregressive fashion (similar in concept to GPT [71] to predict the next symbol in the SMILES sequences by attending over all previously seen symbols. The loss function was the same as with the RNN shown in Eq. 1.

The hyperparameters were the same for both the transformer encoder architectures. More specifically, each consisted of 4 encoder layers with hidden dimension 512, each with 8 multi-attention heads and finally a feed-forward network of hidden dimension 1024. A dropout of 0.1 was used throughout. Each model was then trained on the GuacaMol training dataset for 5 epochs with a batch size of 128 and the ADAM optimizer with a learning rate of 0.001. A performance comparison of these models to RNNs according to commonly used metrics can be found in Additional file 1: Tables S1-2.

### Reinforcement learning

We will in the following briefly review reinforcement learning strategies for recurrent neural networks in order to embed our methodological changes into context.

RL introduces the paradigm of an episodic task where an agent (here the RNN) decides an action (${\mathbf{a}}_{\mathbf{t}}\in \mathbf{A}$) (here, the next SMILES symbol) at time step $\mathbf{t}$ based on interaction with an environment which informs the agent on the current state (${\mathbf{s}}_{\mathbf{t}}\in \mathbf{S}$) (here, the SMILES string) and corresponding reward (${\mathbf{r}}_{\mathbf{t}}$) (here, computed at the end of the episode (${\mathbf{R}}_{\mathbf{T}}\in [0,1]$) by the scoring function) in a Markov Decision Process [72]. Different RL strategies can then be used to describe how to navigate this landscape. These usually fall into one of two categories: value-based strategies focus on estimating the value of an action given a particular a state (or value of being in a state) and selecting an action so as to maximize the final estimated return ($G=\sum_{t}^{T}{r}_{t}$), while policy-based RL focusses on identifying the best policy (${\varvec{\pi}}$) for selecting actions without necessarily consulting a value function to estimate the absolute value of that state/action.

The practical nature of SMILES-based RNN molecule generation complicates the use of value-based RL strategies as incomplete SMILES generated at different time steps do not always result in a valid molecule for which a reward can be assigned. In contrast, policy methods do not require a reward for each action/state and as such are typically used in this setting [4, 5, 22]. Furthermore, as discussed by Olivecrona et al. [4], an RNN is first trained on a large dataset of example molecules which effectively constitutes a prior policy for molecule generation, thus only small changes to the prior policy may be needed.

As a simple baseline strategy, we implemented REINFORCE [73] which is also used in [5, 41]. This is an ‘all-actions’ policy-based method because the policy update only requires a sum over all actions and the return for the whole episode (final molecule)—important due to potentially invalid partial smiles during generation. The loss function is described in Eq. 2 where it can be interpreted as a scaling of the policy (here the negative log likelihood, also described in Eq. 1) by the reward given to the complete molecule (${\mathbf{R}}_{\mathbf{T}}$).

2 $$L\left(\theta \right)=\left[-\sum_{t=0}^{T}logP({a}_{t}|{s}_{t-1})\right]{R}_{T}$$

**Fig. 2:**
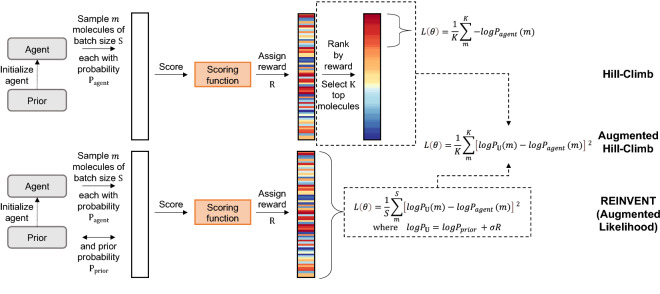
Depiction of the REINVENT, Hill-Climb (HC) and Augmented Hill-Climb (AHC) optimization algorithms and subsequent loss functions ***L*** as parameterized by network parameters ***θ***. AHC is a hybrid algorithm that combines elements of REINVENT and HC

In this work, we implemented REINVENT [4, 58] (depicted in Fig. 2) which is a popular strategy used in the literature, and the strategy we used in our previous work [52]. REINVENT is a REINFORCE type strategy that explicitly regularizes optimization by adding a prior policy to the loss function. This prior policy is derived by computing the negative log likelihood from a fixed copy of the initially trained RNN (the prior). This regularization ensures that the RNN being optimized (the agent) maintains what was initially learnt by the prior i.e., how to generate valid SMILES corresponding to the training distribution. A combination of this prior policy and scaled reward (scaled by scaling coefficient sigma (**σ**)) is then used to define an augmented likelihood, as shown in Eq. 3. This augmented likelihood then acts as a target policy for the agent and the loss function is now defined as the difference between the agent policy and target policy, shown in Eq. 4. Note that we have replaced $-\sum_{t=0}^{T}logP({a}_{t}|{s}_{t-1})$ by the equivalent term $logP(A)$.

3 $${logP}_{\mathbb{U}}\left(A\right)={logP}_{prior}\left(A\right)+ \sigma {R}_{T}$$

4 $$L\left(\theta \right)= \left[{logP}_{\mathbb{U}}(A)-{logP}_{agent}(A)\right]{ }^{2}$$

Recently a strategy was proposed that offered modest performance improvement over REINVENT called ‘best agent reminder’ (BAR) [20], although this was implemented on a graph-based generative model. We have implemented it for an RNN using the same principle to compare it to the other strategies used here as another baseline strategy. This mechanism keeps track of the best agent so far, updating it periodically. During optimization, a batch of molecules **m** (of batch size **S**) is sampled from both the current agent (${M}_{agent}$) and best agent (${M}_{best}$), to serve as a reminder of high scoring molecules. Although the loss function is the same as Eq. 4 for the respective agents, the loss weighted average is taken across agents scaled by **α**, as shown in Eq. 5. This effectively acts to minimize the agent policy difference to the ‘best agent optimal policy’ and the ‘prior optimal policy’, scaled by α.

5 $$L\left(\theta \right)=\frac{\left(1-\alpha \right)}{S}\sum_{m\in {M}_{agent}}\left[{logP}_{{\mathbb{U}}_{prior}}\left(A\right)-{logP}_{agent}\left(A\right)\right]{ }^{2}+\frac{\alpha }{S}\sum_{m\in {M}_{best}}\left[{logP}_{{\mathbb{U}}_{best}}(A)-{logP}_{agent}(A)\right]{ }^{2}$$

Hill-Climb (HC) [41] is an alternative policy-based strategy benchmarked in [22, 36] that shows state-of-the-art or near state-of-the-art performance. HC can also be interpreted as a form of iterative fine-tuning (where fine-tuning molecules are selected by the scoring function rather than e.g., known activity against a certain target). The agent RNN first samples a batch of molecules, and then the RNN is fine-tuned using the same loss function as Eq. 1 but using only the top ***k*** molecules from the batch, as ranked according to some reward assigned to each molecule. This algorithm is depicted in the top part of Fig. 2.

As the RL strategies REINFORCE and HC are not explicitly regularized (as they are in REINVENT, BAR and the method presented here, AHC), cost terms can be added to the loss function to achieve regularization. This step is important in practice to maintain some similarities to the training distribution but also to not catastrophically forget chemical principles which will result in invalid structures (due to valency errors etc.). To assess the effectiveness of this, we evaluated the addition of the Kullback–Leibler (KL) divergence between the prior and agent scaled by a scaling coefficient ***λ***, as shown in Eq. 6 and as implemented in [41, 74]. This adds a constraint to ensure the distribution of agent action probabilities does not differ too much from the distribution of prior action probabilities.

6 $$C(KL)= {\lambda }_{KL}{\mathbb{E}}\left[\sum_{t=0}^{T} \sum_{{a}_{i}\in A}{P}_{agent}({a}_{i}|{s}_{t-1})log\frac{{P}_{agent}({a}_{i}|{s}_{t-1})}{{P}_{prior}({a}_{i}|{s}_{t-1})}\right]$$

Unless otherwise specified, the hyperparameters used for the different RL strategies are those reported in each individual study. They are listed in Additional file 1: Table S3. The number of RL update steps was adjusted to result in an approximately equal number of molecules sampled during training. Hill-Climb* was included to investigate the effect of a smaller batch size in line with AHC.

### Augmented Hill-Climb

In this work, we define a new strategy we call Augmented Hill-Climb (AHC), depicted in Fig. 2 with its constituent parts shown at the top (for HC), and bottom (for REINVENT). This strategy is a simple hybrid between the HC and REINVENT strategies where the loss is calculated as in REINVENT (by defining the augmented likelihood) but only on the top ***k*** molecules, as ranked by reward as in HC. The rationale behind this strategy is based on practical limitations of the REINVENT loss function: when low scoring molecules (${R}_{T}\to 0$) are sampled the score contribution goes to zero and ${logP}_{\mathbb{U}}\left(A\right)\approx {logP}_{prior}(A)$. In this situation, as the loss function (Eq. 4) is a distance, the agent policy will, in-fact, trend back towards the prior policy which may negate useful learnings. This situation of low scoring molecules being present will occur especially either early in the learning process or when a difficult or highly constrained scoring function is used. Therefore, the heavy regularization effect of low scoring molecules significantly contributes to slow learning in these situations. In turn, focussing learning only on the high scoring molecules (${R}_{T}\to 1$) will improve learning. It is worth noting that, high scoring molecules are still regularized by the prior policy, as shown in Eq. 3, ensuring prior learnings are not ‘forgotten’.

### Diversity filters

Applying diversity filters (DFs) is a way of penalizing the reward for an associated molecule based on the molecular similarity to previously generated molecules resulting in diminishing returns for exploitation, therefore encouraging exploration outside of local minima. Blaschke et al*.* [75] introduced several DFs for RNN molecule generation based on different measures of similarity including *compoundsimilarity* (Tanimoto similarity of compound ECFP [76] fingerprints), *identicalmurckoscaffold* (matching Bemis-Murcko scaffolds [77]), *identicaltopologicalscaffold* (matching Bemis-Murcko scaffolds with all atoms treated as carbon atoms and bonds as single bonds) and *scaffoldsimilarityatompair* (Tanimoto similarity of scaffold atom pair fingerprints [78]). More specifically, if generated molecules receive a high enough score by a scoring function (*minimum score threshold*) then the molecules are added to bins based on similarity as defined by any of the above-mentioned DFs. Molecules assigned to a bin are subsequently penalized according to a binary, sigmoid or linear score transformation (*output mode*) based on the maximum allowed *bin size*. Blaschke et al. [75] showed that DFs result in increased diversity of de novo compounds as measured by an increased number of analogues to known molecules.

In addition, we investigated the use of the following DFs:

1. *unique—*a simple DF to serve as a baseline. This DF transforms a molecule’s score to zero if the molecule is non-unique.
2. *occurrence*—This DF linearly penalizes non-unique molecules based on the number of previous occurrences, which acts as a more lenient version of the *unique* DF. The score is transformed according to the number of previous occurrences ($\mathbf{O}\mathbf{c}\mathbf{c}$) beyond an allowed tolerance ($\mathbf{T}\mathbf{o}\mathbf{l}$) until a hard threshold is reached, referred to as the buffer ($\mathbf{B}\mathbf{u}\mathbf{f}\mathbf{f}$). This is shown in Eq. 7.
  7 $${\text{Filtered reward}} = \left\{ {\begin{array}{*{20}c} {{\text{R}}_{{\text{T}}} \times \frac{{{\text{Occ}} - ({\text{Tol}} + {\text{Buff}})}}{{{\text{Tol}} + {\text{Buff}}}}} & {{\text{if}}} & {{\text{Tol < Occ}} < {\text{Buff}}} \\ {{\text{R}}_{{\text{T}}} } & {{\text{if}}} & {{\text{Occ}} \le {\text{Tol}}} \\ 0 & {{\text{if}}} & {{\text{Occ}} \ge {\text{Buff}}} \\ \end{array} } \right.$$
3. *scaffoldsimilarityecfp*—This DF is a modification to *scaffoldsimilarityatompair* introduced in [75] that uses the same parameters except for measuring similarity based on the Tanimoto similarity of the Bemis-Murcko [77] scaffold ECFP4 [76] fingerprints as implemented by RDKit [79].

The DFs and parameters used in this work (i.e., DF1, DF2 and DF3) for tasks other than the parameter search in Experiment 3 are shown in Additional file 1: Table S4.

### Scoring functions and benchmarking tasks

Several scoring functions were used in this work to guide optimization and benchmark RL strategies. These are summarized in Table 1 and are described in more detail in the subsequent sections. All scoring functions were implemented using the MolScore platform [60].

**Table 1:** Summary of all objectives/tasks used in this work and for which experiment (see Fig. 1)

Experiment	Aim	Objective type	Objective target	Performance measure
1	Compare REINVENT and AHC for varying values of σ	Docking	DRD2	Docking score & uniqueness
2	Compare REINVENT and AHC against different target systems	Docking	DRD2	Docking score & uniqueness
		Docking	OPRM1	Docking score & uniqueness
		Docking	AGTR1	Docking score & uniqueness
		Docking	OX1R	Docking score & uniqueness
3	Investigate and identify optimal DF and respective parameters for use with AHC	Similarity	Aripiprazole	Tanimoto similarity, uniqueness & wall time
		Isomer	C_11_H_24_	Isomer score, uniqueness & wall time
		Similarity & PhysChem (MPO)	Osimertinib	MPO score, uniqueness & wall time
4 & 5	Benchmark AHC to other commonly used RL strategies	PhysChem	Heavy atoms	# Heavy atoms, validity, uniqueness & wall time
		Similarity	Risperidone	Tanimoto similarity, validity, uniqueness & wall time
		Activity	DRD2	Predicted activity, validity, uniqueness & wall time
		Docking	DRD2	Docking score, validity, uniqueness & wall time
		Dual activity (MPO)	DRD2 & DRD3	Average predicted activity, validity, uniqueness & wall time
		Selectivity (MPO)	DRD2 > DRD3	Average predicted activity, validity, uniqueness & wall time

### Target preparation and docking tasks

Four different targets were used to setup molecular docking scoring functions to evaluate docking score optimization by RNNs in combination with RL strategies (Experiments 1, 2, 4 and 5 in Fig. 1). The four targets and corresponding x-ray crystal structures used in the docking tasks were D_2_ (DRD2, PDB: 6CM4 [80]), µ (OPRM1, PDB: 4DKL [81]), AT_1_ (AGTR1, PDB: 4YAY [82]) and OX_1_ (OX1R, PDB: 6TO7 [83]) receptors.

All target crystal structures were first prepared using Schrodinger Protein Preparation Wizard [84] using default parameters which included: addition of protein and ligand hydrogens (pH 7 $\pm$ 2, Epik [85]), optimization of hydrogen bond networks (pH 7, PROPKA [86]), restrained minimization using the OPLS3e force field [87], and removal of waters (except for OPRM1 which performed better retrospectively with crystallographic waters, data not shown). A default grid was defined using the respective co-crystallized ligands as the centre except for OX1R which had additional positional restraints defined based on consensus sub-pocket occupation by the following overlayed co-crystallized ligands, Suvorexant (PDB: 6TO7), Filorexant (PDB: 6TP6), Daridorexant (PDB: 6TP3), GSK1059865 (PDB: 6TOS), ACT462206 (PDB: 6TP4), Compound-16 (PDB: 6TQ4), Compound-14 (PDB: 6TQ6), EMPA (PDB: 6TOD) and Lemborexant (PDB: 6TOT) [83].

Before docking, ligands were prepared using Schrodinger LigPrep [88] to enumerate unspecified stereocentres, tautomers and protonation states, with up to 8 variants generated per molecule, based on a pH range of 7 $\pm$ 1. Variants were then docked using Glide-SP [89] with default settings, except for OX1R where docked poses were only accepted if they satisfied four out of five grid constraints. The lowest (i.e., best) docking score achieved by any molecule variant was returned as the final docking score. Docking score was normalized between the values of 0 and 1 based on all previously observed docking scores.

Retrospective performance was assessed by docking known active and inactive molecules extracted for each human target from the ExCAPE-DB [90]. When more than 10,000 labelled molecules were present, a random subset of 10,000 molecules was taken. To better represent de novo molecules docked which adhere to property constraints imposed by MOSES^n^, molecules above 500 Da were filtered out, stereo information removed, and any resulting duplicates removed. The final number of downloaded and docked molecules is shown in Additional file 1: Table S5. Classification accuracy, precision and recall were assessed by varying docking score decision thresholds (Additional file 1: Figure S1). In each case a threshold corresponding to ~ 80% precision was identified, i.e., ~ 80% of molecules below this threshold are true actives retrospectively. The typical recall of true actives at this level was ~ 10–30%.

### Diversity filter parameter optimization tasks

To investigate the effect of DF and parameter choice, less computationally expensive scoring functions were required than docking. Therefore, three diverse tasks from the GuacaMol benchmarking suite [22] were chosen and re-implemented according to the original work [22]. The goal the Aripiprazole similarity task is to optimize similarity to Aripiprazole beyond a similarity threshold in order to generate as many similar enough compounds as possible. The goal of the C_11_H_24_ isomer task is to generate all 159 molecules with a molecular formula of C_11_H_24_, a task involving a more limited pool of molecules. The goal of the Osimertinib MPO task is to optimize similarity to Osimertinib to a certain extent (molecules are penalized if too close) and that both lipophilicity and polarity are within a suitable range. The performance of DF parameters was measured by the area under the training curve of three different endpoints: uniqueness (number of unique molecules generated, a proxy of chemical space explored and symptom of mode collapse), goal (the score returned by the scoring function/s) and run time (a practical measure to identify if some DFs are slower to compute).

### QSAR model training

Active and inactive molecules against DRD2 and against DRD3 were extracted from ExCAPE-DB [90]. This corresponded to 4609 and 2758 active molecules and 343,026 and 402,524 inactive molecules respectively. A further unique subset was defined for each target by excluding molecules with measured activity against the other target to ensure no domain overlap between DRD2 and DRD3 models for the dual and selective tasks, resulting in in 2282 and 373 active molecules and 5161 and 64,717 inactive molecules for DRD2 and DRD3 respectively. To tackle data imbalance, a maximally diverse selection of 5000 inactive molecules were selected for DRD2 and DRD3, respectively, via a MaxMin algorithm [91] on ECFP4 fingerprints with 2048 bits, implemented in RDKit. Three random forest (RF) classification models were trained to predict probability of activity (with 100 estimators, max depth of 15 and minimum leaf sampled of 2), one on all DRD2 data with the diverse inactive subset and two on DRD2 and DRD3 unique data with diverse inactive subsets, all implemented in scikit-learn [92]. In each case model performance was estimated by stratified, active cluster split (inactive molecules were split randomly due to being a maximally diverse selection) fivefold cross-validation with GHOST decision threshold identification [93] resulting in the performance shown in Additional file 1: Figure S2.

### Reinforcement learning strategy benchmark tasks

Six further tasks of varying practical difficulty were used to benchmark the different RL strategies at three levels of objective complexity:

1. *# Heavy atoms*—This ‘easy’ task aims to maximize the number of heavy atoms in a molecule calculated by RDKit [79]. This is similar in concept to maximizing penalized LogP [13] and QED [94] which has been shown to be trivial by some generative models [13, 15, 95]. Although we stress that this probes the RL strategy’s ability to extrapolate beyond the training dataset (which contains molecules with a limited number of heavy atoms), rather than as a measure of good performance. However, this task is irrelevant to real drug discovery objectives.
2. *Risperidone similarity*—This ‘easy’ task aims to maximize the Tanimoto similarity to Risperidone (a DRD2 inverse agonist and co-crystallized ligand in PDB: 6CM4) according to ECFP4 fingerprints with a bit length of 1,024 (as implemented in RDKit). While this tests the ability to move to a precise region of chemical space, it is unlikely to be relevant as a real drug discovery objective due to lack of novelty. The ability of generative models to easily maximize such tasks has been shown in benchmark studies [22].
3. *DRD2 activity*—This ‘medium’ task aims to maximize the QSAR predicted probability of activity against DRD2 (Eq. 8). This task is representative of a real objective during early-stage hit finding, providing that known ligand data is available. Maximization of these tasks are often achieved by generative models [4, 5, 15] but is a more scientifically complex objective than molecular similarity.
  8 $$DRD2\,active = P_{{RF}} (DRD2)$$
4. *DRD2 docking score*—This ‘medium’ task aims to minimize the Glide-SP docking score (predicted binding affinity) against DRD2. This task is representative of a real objective during early-stage hit finding, providing that a crystal structure or homology model is available. It was implemented as described above with the exception that molecules were instead prepared by enumerating up to 16 stereoisomers using RDKit [79] and then conducting protonation using Epik (pH 7.4) to only protonate the most abundant state per stereoisomer. This task has been successfully optimized by generative models in some cases [47] but proven difficult in others [35].
5. *DRD2-DRD3 dual*—This ‘hard’ task aims to maximize the QSAR predicted probability of activity against both DRD2 and DRD3 (Eq. 9). This task is representative of real drug discovery projects requiring polypharmacological activity, providing that ligand data for both is available. This constitutes a multi-objective optimization problem which has proven more difficult for generative models with an increasing number of constraints [15, 47, 96].
  9 $$DRD2 - DRD3\,dual = \frac{{P_{{RF}} \left( {DRD2_{{unique}} } \right) + P_{{RF}} (DRD3_{{unique}} )}}{2}$$
6. *DRD2/DRD3 selective*—This ‘hard’ task aims to maximize the QSAR predicted probability of selective activity against DRD2 over DRD3 (Eq. 10). This is representative of real drug discovery projects that must avoid off-target effects for toxicity or efficacy reasons, providing that ligand data for both is available. Similar to dual inhibition, multi-objective optimization problems are more difficult for generative models to optimize against [15, 96].
  10 $$DRD2/DRD3{\mkern 1mu} \,selective = \frac{{P_{{RF}} (DRD2_{{unique}} ) + (1 - P_{{RF}} (DRD3_{{unique}} )}}{2}$$

## Results and discussion

### Optimization of DRD2 docking score by Augmented Hill-Climb compared to REINVENT

Optimization ability and sample-efficiency was assessed using the procedure described in Methods (Experiment 1, Fig. 1). Specifically a RNN was trained on the MOSES^n^ dataset [23, 52], an agent was initialized which then underwent RL updates to optimize the docking score of de novo molecules against DRD2. The REINVENT strategy and docking protocol was identical to our previous work [52].

**Fig. 3:**
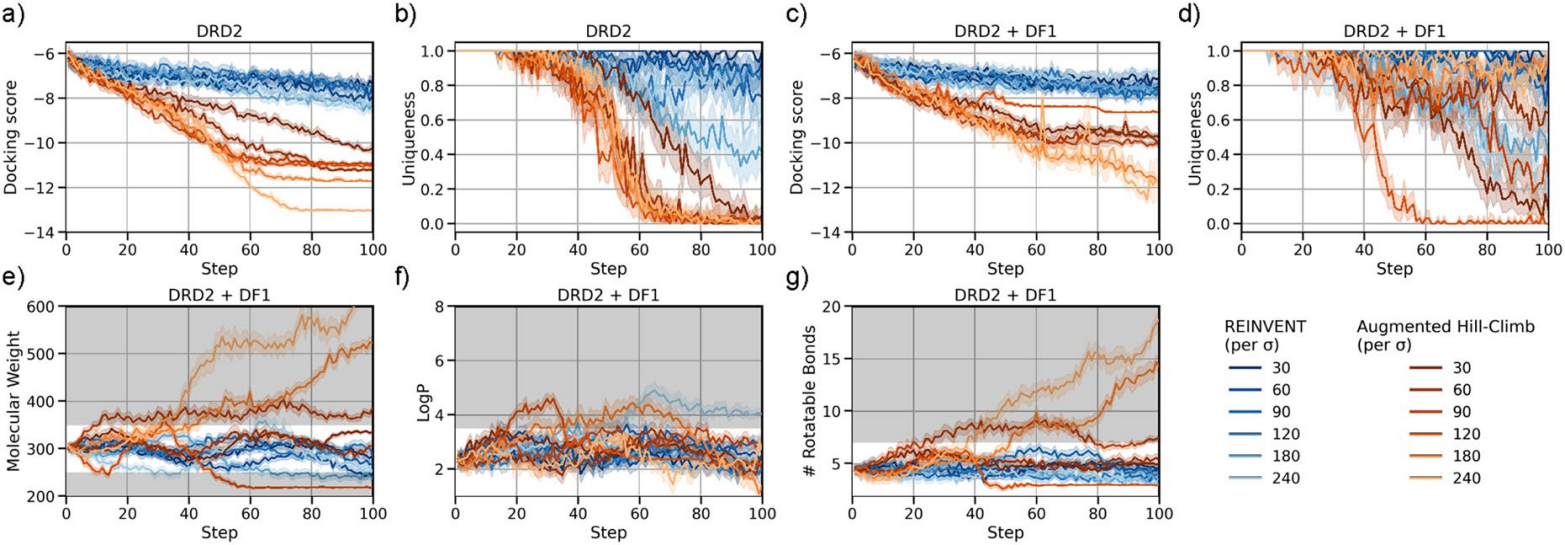
Comparison between REINVENT and Augmented Hill-Climb learning strategies to optimize DRD2 docking scores at varying levels of σ. (**a**) Augmented Hill-Climb is more efficient at optimizing docking score at all levels of σ but (**b**) undergoes increased mode collapse via a drop in uniqueness. (**c**) Docking score optimization can be stabilized and (**d**) mode collapse rescued by applying a diversity filter. (**e–g**) Augmented Hill-Climb in combination with DF1 is more sensitive to changes in σ, this affects the extent to which de novo molecules occupy property space which is not present in the prior training set (grey shaded area) i.e., extrapolation

To increase optimization power, the easiest proposal is to increase the score contribution to the augmented likelihood used by REINVENT by increasing the scalar value σ. The original work [4] had a default value of 60, however, the subsequent update (REINVENT 2.0 [58]) increased this value to 120—suggesting that sample-efficiency was sub-optimal. Therefore, we first varied the value of σ between 30 and 240 and updated an agent for 100 RL steps only (6400 samples), to minimize computational expense. However, as shown in Fig. 3a, we found little improvement in optimization of DRD2 docking scores using this approach with REINVENT. The maximum docking score optimization achieved (best mean score relative prior mean score) was 128% with σ = 60 or 127% with σ = 240, concluding that changing σ values alone did not significantly improve optimization over limited RL updates.

AHC was then implemented in an effort to improve sample-efficiency, while also varying σ and for the same amount of RL updates (Fig. 3a). This consistently led to improved optimization ability for every σ value compared to REINVENT, with a maximum of 205% optimization with σ = 240. In total, we found a 1.39-fold improvement in optimization ability compared to REINVENT averaged across all values of σ. Moreover, AHC required approximately 80 fewer steps to achieve the mean docking score achieved by REINVENT over 100 steps, evidencing a large improvement in sample-efficiency. However, learning was stifled by a drop in uniqueness observed (Fig. 3b) i.e., AHC was more prone to mode collapse.

To address the mode collapse, a diversity filter (DF1) [75] was applied to both strategies to penalize exploitation and hence encourage exploration. DF1 penalizes the score of any of the top 20% of de novo molecules that were similar to previously generated molecules, a threshold chosen based on the nature of docking-based virtual screening where only the very top ranked molecules are considered. This stabilized learning and rescued the drop in uniqueness in most cases (Fig. 3c and d). With DF1, AHC evidenced a σ-averaged 1.45-fold improvement compared to REINVENT (with a maximum optimization of 192% at σ = 180 for AHC, compared to 119% at σ = 180 for REINVENT). Similar to without the DF1, AHC still required 80–90 fewer RL steps to achieve a mean docking score achieved by REINVENT over 100 steps.

Although increasing the σ value increases the score contribution to the loss, it also decreases the prior contribution and thus decreases regularization during optimization. As such, we expect that larger values of σ result in further extrapolation outside the domain of the training set and prior, which is the aspect of the generated molecules we analysed next. Figure 3e–g show the properties of de novo molecules generated during optimization and the property space not occupied by molecules in the MOSES^n^ dataset—serving as a proxy to assess extrapolation. AHC in combination with DF1 is more sensitive to changes in σ, where larger values of σ do result in extrapolation into property space that is absent in MOSES^n^, more so than REINVENT in combination with DF1. In practice, this extrapolation can be both favourable (by identifying novel chemical space) or unfavourable (by enabling exploitation of scoring function flaws, such as molecules with more heavy atoms providing better docking scores simply due to the additive nature of docking scoring functions [97]). In either case, it is advantageous to have greater control over this trade-off, which is achieved as variations in σ show more impact for AHC over REINVENT. Importantly, AHC still improves 1.47-fold over REINVENT at σ = 60, where both strategies are sufficiently regularized and maintain the property space as defined by MOSES^n^.

Despite improvement in the optimization ability by AHC, it is irrelevant if the resulting de novo structures are invalid or implausible (e.g., incorrect valences, unstable or idiosyncratic functional groups or strained ring systems). The chemistry generated by RNNs has been evaluated previously [3, 23, 33, 98, 99] and has usually been considered reasonable with respect to overall topology, fragments, substructures and property space. On the other hand, a comparison of chemistry between AHC and REINVENT is complicated by the scoring function and its suitability for an objective e.g., greater optimization may actually lead to unreasonable chemistry due to scoring function exploitation rather than as a function of the RL strategy. We note that this analysis of scoring function suitability is out of the scope of this work but we aim to cover this in future work. On the other hand, the REINVENT strategy has been shown to maintain similar chemistry to the prior RNN [4, 52, 53, 75]. Therefore, we visually compared some of the top molecules generated at different values of σ, shown in Additional file 1: Figure S3. At lower values of σ (30–120) and with no regard for prior knowledge of DRD2 ligand topology, the molecules are mostly indistinguishable as to which RL strategy was used. With regard for DRD2, both strategies learn to generate benzyl/bicyclic moieties with a protonatable amine above. This chemotype is consistent with the co-crystallised inverse agonist risperidone [80] and required interactions to D114^3x32^ for ligand activity [99–102], where the cyclic moiety would sit deep in the hydrophobic sub-pocket and the amine would form a salt bridge with D114^3x32^. The only difference between the RL strategies appears to be the better docking scores achieved by AHC. However, as σ increases (180–240), de novo molecules are clearly much larger and therefore exploiting the additive nature of the docking scoring function [97]. This corroborates the observation of extrapolation into restricted property space seen in Fig. 3e and g, which enables this exploitation. In this scenario added constraints would be necessary in a multi-parameter optimization setting, such as also defining a suitable molecular weight range as this constraint is no longer imposed by the prior dataset. We believe these results highlight the balance that is required in the trade-off between regularization and optimization, which is better achieved by AHC than REINVENT.

### Optimization of docking scores for multiple GPCR targets

**Fig. 4:**
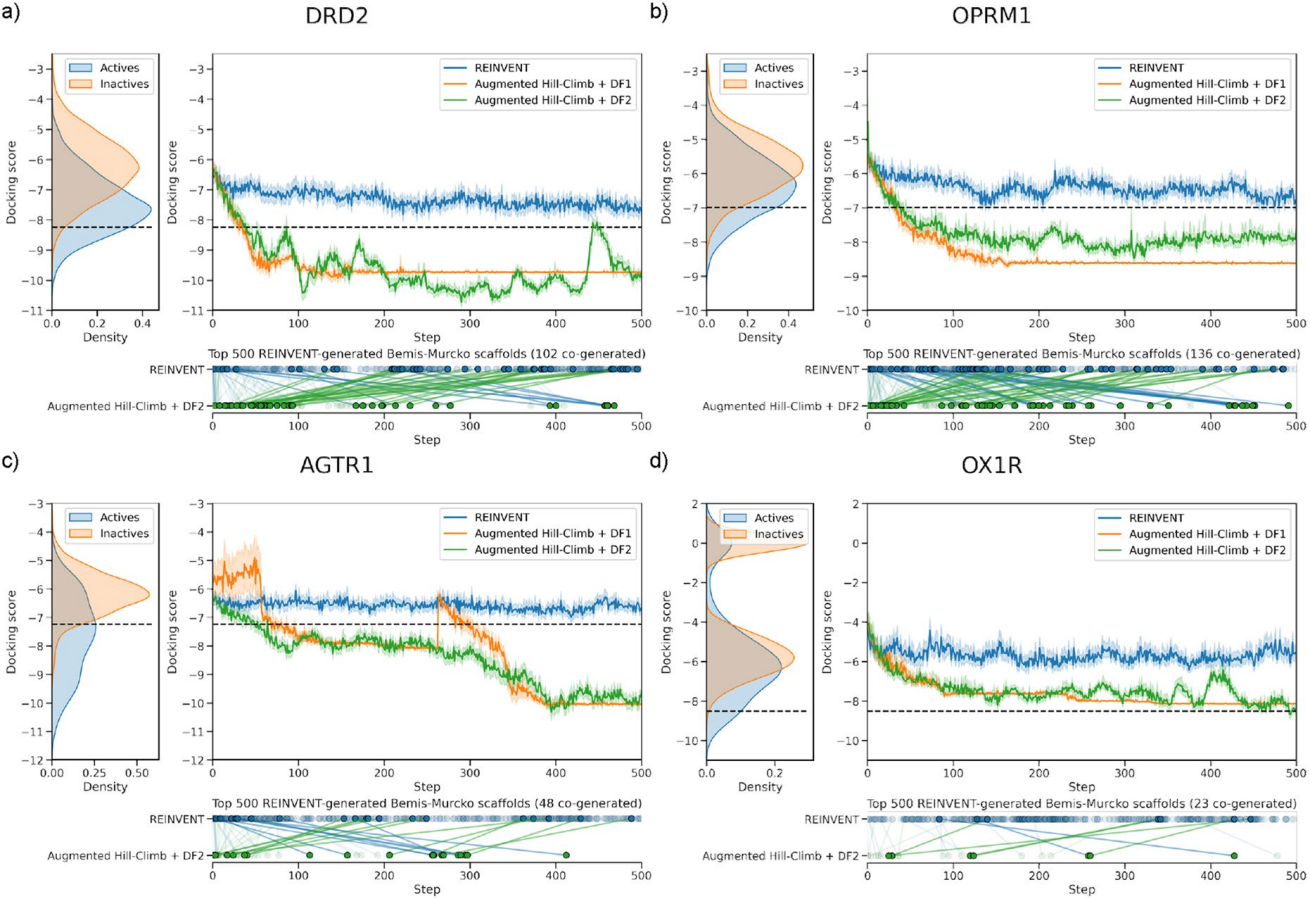
Improved learning efficiency of Augmented Hill-Climb against four targets: (**a**) DRD2, (**b**) OPRM1, (**c**) AGTR1 and (**d**) OX1R. (top left panel) Distribution of known active and inactive molecule docking scores. (top right panel) Optimization of de novo molecule docking score via reinforcement learning. (bottom right panel) The top 500 REINVENT generated scaffolds with the corresponding time of generation by REINVENT or by Augmented Hill-Climb (in combination with DF2) if co-generated. Blue lines represent scaffolds generated by REINVENT first and green lines generated by Augmented Hill-Climb (in combination with DF2) first. Scaffolds with a difference in generation time of < 100 RL updates are more transparent. Augmented Hill-Climb in combination with DF2 shows improved learning efficiency compared to REINVENT and optimizes past a docking score threshold corresponding to a retrospective classification precision of 80% (black dashed line) in all cases

Previously, we used REINVENT to optimize the docking score against other GPCR targets (DRD2, OPRM1, AGTR1 and OX1R) over the course of 3000 RL updates, the first 500 updates of which are shown in Fig. 4. DRD2 [80] (same data as previously published [52]) contains a deep hydrophobic sub-pocket and requires a salt bridge interaction with D114^3x32^ for ligand activity. OPRM1 [81] similarly forms a salt bridge interaction via D147^3x32^ (a structurally conserved position in aminergic receptors [100, 101]) but with a more open pocket than DRD2. AGTR1 [82] requires important salt bridge and hydrogen bond interactions to R167^4x65^ (e.g., via acidic tetrazole of co-crystallised ligand ZD7155) as well as hydrogen bonds to Y35^1x39^ on the opposite side of the pocket. Meanwhile OX1R [83] contains four well defined hydrophobic sub-pockets and sometimes a hydrogen bond to N318^6x55^ and water mediated hydrogen bond to H344^7x38^, ligands are found to adopt a horseshoe conformation via π-stacking to satisfy these sub-pockets as in the co-crystallised ligand suvorexant. The first two targets’ respective docking scores were able to be minimized similarly (Fig. 4a and b), while the latter two targets’ respective docking scores were more challenging and showed little minimization (Fig. 4c and d) (especially with respect to the distribution of docking scores for known actives). This suggests that the docking score optimization ability of REINVENT was system dependent or that the MOSES^n^ dataset used for RNN pretraining did not contain chemistry amenable to minimizing the docking score for these systems.

**Fig. 5:**
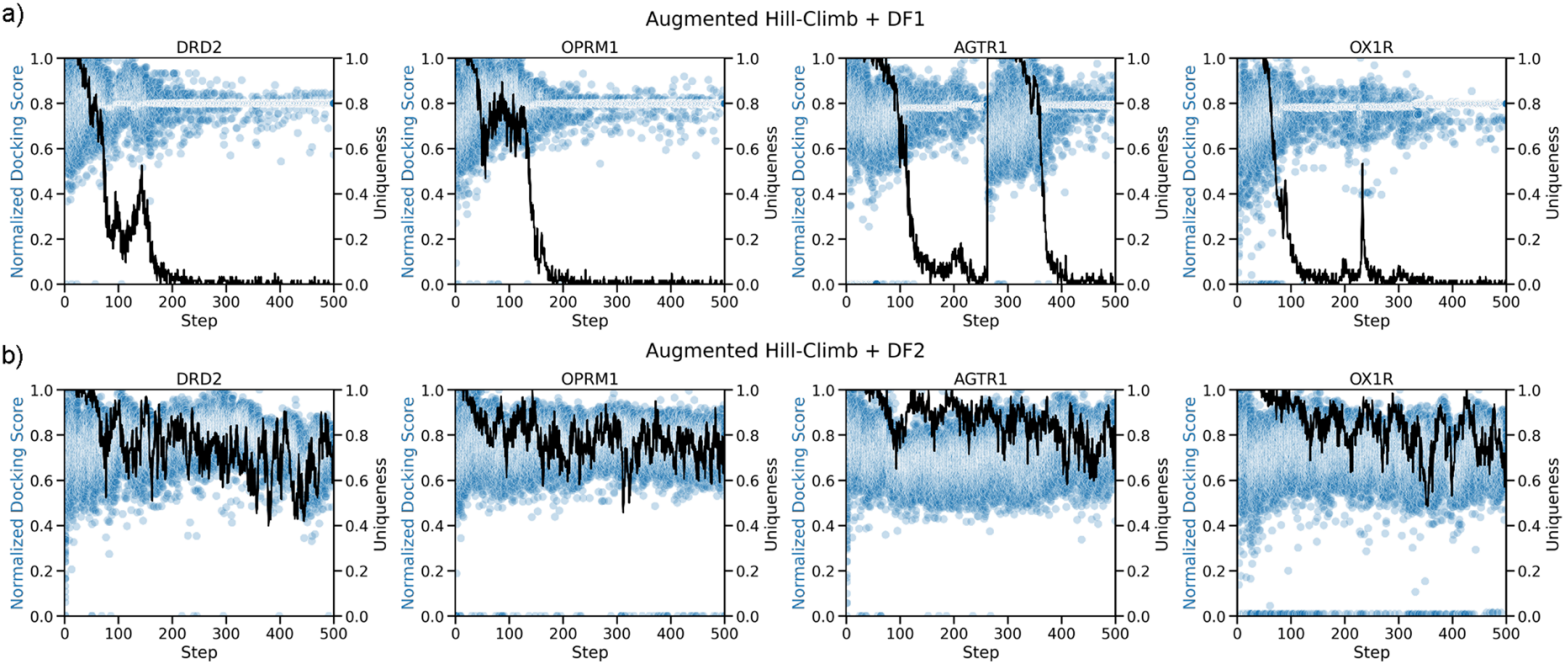
Optimization of normalized docking score and uniqueness during optimization across three targets. (a) With diversity filter 1 (DF1), docking score converges to the minimum score threshold (0.8) of the DF and model undergoes mode collapse seen by an associated drop in uniqueness. (b) With diversity filter 2 (DF2), no convergence is observed, and uniqueness maintains relatively high. This is due to a lower minimum score threshold (0.5) and softer penalization scheme

Given the improved optimization power of AHC in combination with DF1 seen with fewer RL updates against DRD2, AHC in combination with DF1 was compared to these REINVENT results to see if improvement was consistent over 500 RL updates and for different GPCR targets (Experiment 2, Fig. 1). For every target, AHC in combination with DF1 (Fig. 4) resulted in faster and further minimization of the docking score. For reference, the 80% retrospective precision threshold was surpassed within 100 RL updates in all cases except for the particularly challenging OX1R. However, docking score plateaus for AHC in combination with DF1 in later stages of training. This plateau signals mode collapse as uniqueness drops, similar to training without a DF as shown in Fig. 3a. Interestingly, a convergence of the normalized docking score towards the minimum score threshold of the DF occurs, and uniqueness then drops for all targets (Fig. 5a). It appears that the model learns to generate molecules with a score just below the minimum score threshold to avoid DF penalization and is thus vulnerable to mode collapse as observed without the DF (Fig. 4a, b).

Therefore, we conducted a search of DFs and hyperparameters to identify a more optimal configuration that would successfully and robustly rescue mode collapse (Experiment 3 in Fig. 1) (see next subsection). This led to the design of DF2 which differed from DF1 by having a lower minimum score threshold of 0.5 instead of 0.8, linear penalization output mode instead of binary, and larger bin size of 50 instead of 25. Using DF2 we re-ran the previous experiment (Experiment 2 in Fig. 1) on the four targets as before, shown in Fig. 4. The change in DF stabilized learning over the full length of training while still resulting in similar optimization of docking score. Moreover, there was no convergence of normalized docking score to the minimum score threshold and thus uniqueness stayed relatively high (Fig. 5b). To gain a quantitative understanding of improvement in sample-efficiency, Table 2 compares the number of steps (and samples) required by AHC in combination with DF2 and REINVENT to reach various thresholds during optimization. This shows that the largest improvement over REINVENT is made early, where AHC in combination with DF2 requires 19.8-fold fewer training steps until the mean surpasses 120% optimization, however, both strategies sample a single molecule with a docking score exceeding this threshold within the first batch. Meanwhile, AHC in combination with DF2 took 71.8-fold fewer samples than REINVENT until a molecule surpassed 160% optimization. At 180% and 200% optimization, REINVENT only sampled molecules surpassing the threshold for OX1R and thus fold-improvement could not be calculated, however a minimum estimate is shown based on the maximum number of training steps or samples generated. On average, AHC in combination with DF2 required 7.4-fold fewer training steps and 45.5-fold fewer samples across all targets and all optimization thresholds.

**Table 2:** Number of steps taken before the mean exceeds certain internal and external thresholds (earliest sample exceeding threshold is shown in brackets)

	Threshold	Number of steps required for optimization beyond prior at a given threshold					Number of steps required for optimization beyond external thresholds		
		120%	140%	160%	180%	200%	Inactive mean	Active mean	80% precision threshold
DRD2	REINVENT	> 500 (15)	> 500 (685)	> 500 (22,292)	> 500 (> 32,000)	> 500 (> 32,000)	1 (1)	163 (15)	> 500 (15)
	Augmented Hill-Climb + DF2	19 (2)	6 (49)	105 (1248)	> 500 (3009)	> 500 (23,150)	2 (2)	19 (2)	48 (2)
OPRM1	REINVENT	133 (7)	> 500 (868)	> 500 (7663)	> 500 (> 32,000)	> 500 (> 32,000)	4 (2)	80 (4)	> 500 (7)
	Augmented Hill-Climb + DF2	3 (16)	17 (22)	45 (29)	150 (34)	> 500 (2759)	6 (16)	17 (22)	33 (28)
AGTR1	REINVENT	> 500 (25)	> 500 (510)	> 500 (5,596)	> 500 (> 32,000)	> 500 (> 32,000)	1 (2)	> 500 (8)	419 (6)
	Augmented Hill-Climb + DF2	62 (27)	318 (869)	396 (3,404)	> 500 (5,207)	> 500 (27,979)	2 (1)	62 (27)	46 (2)
OX1R	REINVENT	5 (1)	52 (1)	> 500 (7)	> 500 (142)	> 500 (490)	1 (2)	9 (1)	> 500 (490)
	Augmented Hill-Climb + DF2	9 (1)	15 (2)	31 (2)	87 (31)	382 (557)	2 (1)	14 (2)	494 (557)
Average fold improvement		19.8 (2.5)	11.2 (38.7)	8.3 (71.8)	2.8 (240.6)	1.1 (3.8)	0.5 (1.0)	5.5 (2.1)	9.7 (3.2)

The final row lists the Augmented Hill-Climb in combination with DF2 fold improvement over REINVENT. Where a threshold was not reached within the maximum number of training steps (or samples) it has been annotated as being greater than 500 (or 32,000)

To investigate if similar chemistry was generated by the RL strategies, we identified the top 500 scaffolds generated by REINVENT for each target and plotted at what stage they were first generated by either RL strategy, shown in Fig. 4 (bottom part of each panel of the figure). This shows a general trend where AHC in combination with DF2 tends to generate scaffolds appearing in REINVENT at a later stage much sooner, and scaffolds appearing earlier in REINVENT much later. That is, AHC in combination with DF2 identifies chemistry where the mean docking score has improved more than 100 steps sooner, while early chemistry typically achieved due to batch variance more than 100 steps later—likely because of the DF encouraging exploration and re-visiting sub-optimal chemistry.

**Fig. 6:**
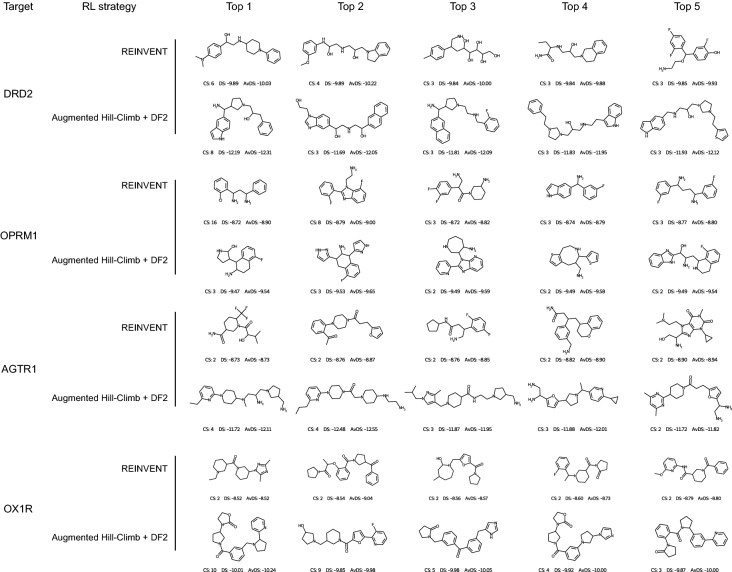
Centroid of the 5 largest clusters for the top 100 molecules according to docking score against DRD2, OPRM1, AGTR1 and OX1R receptors. Cluster size (CS), centroid docking score (DS) and the average cluster docking score (AvDS) is annotated below. In each case Augmented Hill-Climb generates clusters with lower (better) docking scores, while maintaining reasonable chemotypes that are indistinguishable to those generated by REINVENT. Note that protonation states, tautomers and stereoisomers are enumerated by the docking protocol (see Methods)

A visual comparison of the centroids of the top 100 compounds for each target for AHC in combination with DF2 and REINVENT is shown in Fig. 6. With disregard to prior knowledge of target ligands and suitability of the scoring function, the quality of chemistry generated is again indistinguishable between the two RL strategies. However, regarding co-crystal ligands and known important residue interactions, the scoring function is not always suitable as shown in the case of AGTR1. Here we can see no acid moieties are generated for AGTR1 by either strategy (Fig. 6) which will be in part due to the docking algorithm targeting only the Y35^1x39^ sub-pocket and out towards the extracellular surface (Additional file 1: Figure S7c) as opposed to the sub-pocket surrounding R167^4x65^ as required for ligand activity [82].

**Fig. 7:**
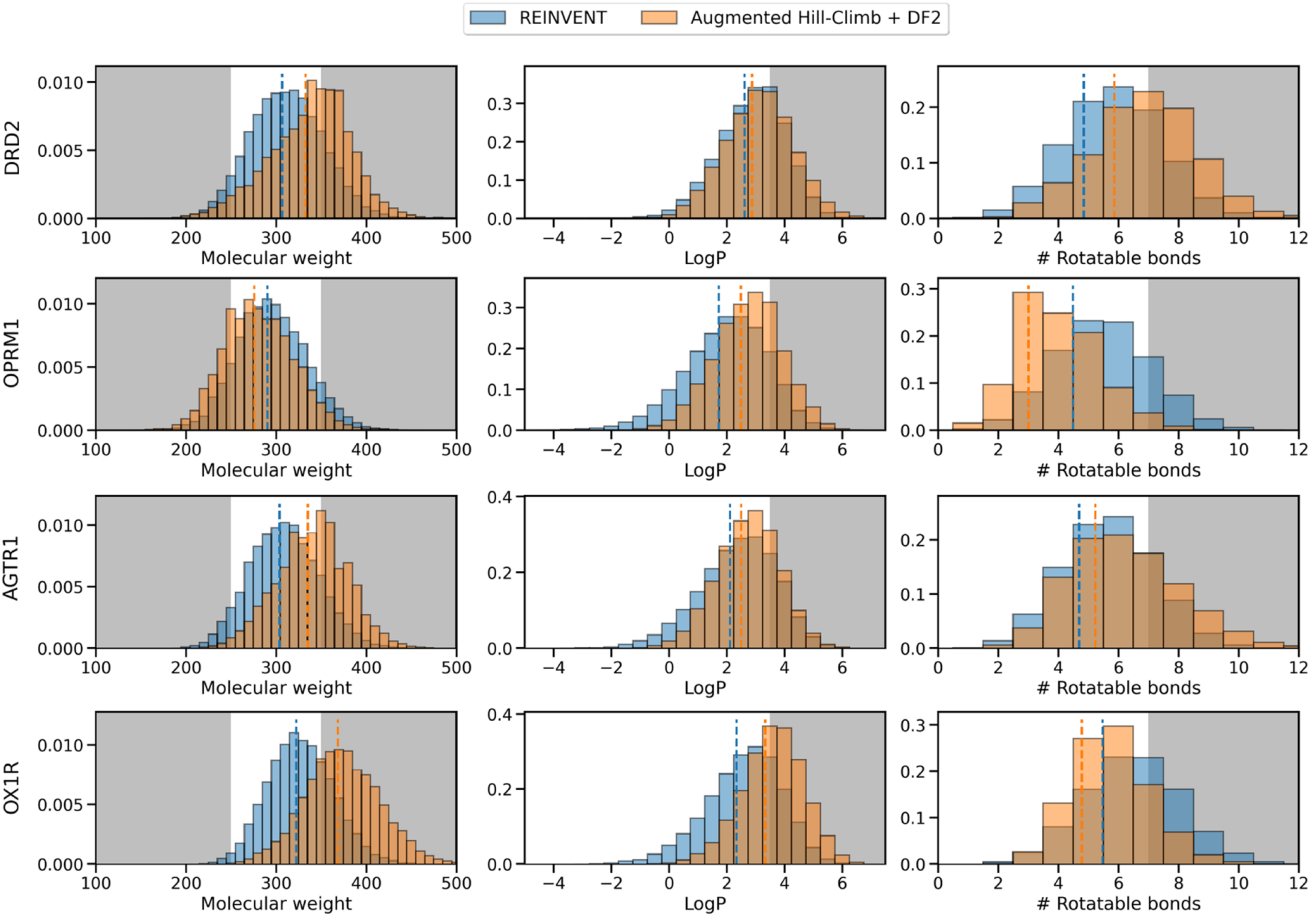
REINVENT compared to Augmented Hill-Climb (in combination with DF2) property space according to molecular weight, LogP and the number of rotatable bonds for molecules optimized to minimize the docking score against four targets. The grey shading indicates property space not represented in the prior training set

In addition, we investigated property space occupied by AHC generated de novo molecules (Fig. 7) which shows that the property space is still maintained (mean remains within training set space) in all cases except for increasing molecular weight seen with OX1R. Here, the mean is slightly above 350 Da which is however consistent with OX1R antagonists [83]. In fact, in some cases (for OPRM1 in the case of molecular weight and number of rotatable bonds, and for OX1R in the case of the number of rotatable bonds) the property space shifts in the opposite direction to that which would be expected by an exploitation of the scoring function. Overall, de novo chemistry is still reasonable and sufficiently regularized by AHC in combination with DF2 and can even be more heavily regularized by reducing σ to 30, yet still outperform REINVENT at all σ values as seen in Experiment 1.

### Effect of Augmented Hill-Climb diversity filter hyperparameters on molecule generation

Given the drop in uniqueness observed in Fig. 5a, we conducted a hyperparameter search to identify optimal diversity filters and respective hyperparameters that best combat mode collapse (Experiment 3 in Fig. 1). We tested DF configurations on three representative objectives taken from the GuacaMol benchmark suite [22] and hence used an RNN architecture, and training regime identical to that implemented in GuacaMol [67] (see Methods), with the exception of using AHC for optimization. This resulted in 825 individual runs across the three objective tasks that were assessed by computing the area under the curve during optimization for uniqueness, score/goal achieved as well as, taking the final run time.

In all cases (Additional file 1: Figures S4-6), we found that a higher minimum score threshold (> 0.5) lead to poorer performance. The higher the minimum score threshold, the fewer molecules the DF is applied to and therefore the closer AHC is to being run without a DF, explaining the drop in uniqueness as observed previously (Figs. 3a, b,  5a). We note that the specific implementation of scoring functions we use has a duplicate lookup function that may result in longer run times if many duplicate molecules are observed, explaining the counter-intuitive increase in run time with less actual DF use (higher minimum score threshold).

With respect to improving uniqueness—the main symptom of mode collapse—lower bin sizes, linear output mode and *compoundsimilarity / scaffoldsimilarityatompair* DFs appear to work best. Lower bin size corresponds to quicker penalization for certain chemotypes, although bin size effect is lesser for the Osimertinib MPO task. In the case of Osimertinib MPO (Additional file 1: Figure S6a), simply penalizing non-unique molecules provides reasonable performance improvement from 0.19 AUC (no DF) to 0.87 AUC. Meanwhile linear output performs best when bin size is greater than 0 (note when bin size is 0 all output modes are effectively binary), suggesting that greater performance is achieved with a more gradual penalization gradient. Lastly, *compoundsimilarity* and *scaffoldsimilarityatompair* DFs slightly outperform all others. We suspect these DFs are a broader measure of similarity than identical scaffolds or *scaffoldsimilarityecfp* resulting in more molecules being identified as similar and therefore penalized. Note that we do not investigate the minimum similarity threshold or fingerprint hyperparameters in this work and leave them as default [75]. Therefore, preventing mode collapse and improving uniqueness typically requires stricter diversity filter parameters that penalize duplicated or similar molecules more easily (although a softer gradient of penalization is preferred).

With respect to the objective score, there was less discrepancy between output modes and the bin size and DF observations effectively reversed. Higher bin sizes and the narrower measures of similarity (*identicalmurckoscaffold* and *scaffoldsimilarityecfp*) showed higher AUCs indicating better performance. We believe these more lenient diversity filter hyperparameters enable AHC more time to associate chemotypes with high rewards resulting in increased objective scores.

Overall, a trade-off is required in choosing DFs and hyperparameters for use in combination with AHC. DF penalization must be strict enough to reliably prevent mode collapse as observed by a drop in uniqueness, yet lenient enough to enable AHC to learn chemotype-reward associations. These observations led us to the design of DF2 which is a compromise between preventing mode collapse and achieving high objective scores.

### Benchmarking Augmented Hill-Climb against other reinforcement learning strategies

The performance of Augmented Hill-Climb was compared to other RL strategies commonly used for language-based RNN de novo molecule generation, namely, REINFORCE [5], REINVENT [4, 58], BAR [20] and Hill-Climb [22], as well as in combination with KL regularization for non-regularized strategies (Experiment 4, Fig. 1). In the interest of standardisation, the prior was trained on the GuacaMol train dataset. The RL strategies were applied to six tasks of varying practical difficulty (see “Methods”). DF2 was used in all cases except for the Risperidone similarity task which uses a lower minimum score threshold of 0 (Additional file 1: Table S4, DF3) due to low reward values observed.

**Fig. 8:**
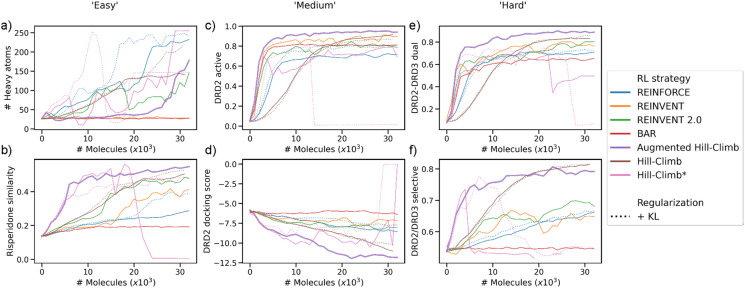
Per-molecule optimization of different RL strategies against different objective tasks of varying difficulty: (**a**) number of heavy atoms, (**b**) Similarity to Risperidone (DRD2 inverse agonist), (**c**) predicted probability of DRD2 activity, (**d**) Glide-SP docking score against DRD2, (**e**) predicted probability of dual activity against DRD2 and (**f**) predicted probability of selective activity towards DRD2 over DRD3. Standard deviation can be seen in Additional file 1: Figure S8. In all cases, except the number of heavy atoms, AHC outperforms all other RL strategies with respect to objective optimization while maintaining validity and uniqueness. Only valid molecules are plotted, therefore gaps seen with HC* denote regions where no valid molecules were generated

The performance of task optimization is shown in Fig. 8. AHC is the most efficient of all RL strategies at all tasks except for maximizing the number of heavy atoms (Fig. 8a). It is particularly better than the other RL strategies during early-stage optimization (e.g., Fig. 8b) and in more difficult objectives (e.g., Fig. 8e, f). AHC even outperforms un-regularized RL strategies. Intriguingly, AHC seems to achieve maximization towards the end of training in the heavy atom task (seen to a lesser extent with REINVENT *2.0*), suggesting it will eventually be able to extrapolate outside the training domain. As AHC uses a considerably smaller batch size than HC and therefore undergoes more frequent network updates, we applied the same batch size to HC to investigate this effect, denoted as HC*. This smaller batch size did in-fact improve sample-efficiency, similar to AHC*,* in early stages of training, but then quickly underwent mode collapse as evidenced by a drop in validity and uniqueness (Additional file 1: Figures S10 and S11). Moreover, KL regularization did not rescue mode collapse in any case, and sometimes worsened performance, suggesting it is not a sufficient regularization method in this context. Interestingly, our re-implementation of BAR performed particularly poorly in most cases except for DRD2 activity (the case study in the original implementation [103]). We propose that the best agent memory in this context may actually inhibit learning without notable improvements in-between updating the ‘best agent’; in effect having two ‘regularizers’ inhibiting learning. As a result, decreasing the ‘best agent’ update frequency (from 5 as originally implemented) may improve performance. Overall, AHC shows a sample-efficiency well beyond other RL strategies for all tasks of practical importance (i.e., excluding the heavy atom task).

The efficiency benefit of AHC is true also by wall time (Additional file 1: Figure S9). To put this practical benefit into greater context, Table 3 shows the CPU hours (i.e., if only 1 CPU was used) required to reach different optimization thresholds for the DRD2 docking score task. AHC is the only strategy able to optimize the mean docking score to 200% that of the initial prior mean docking score within the given time. Moreover, AHC also achieves lower optimization thresholds much quicker, for example, 140% in just 16 h compared to 202 h for REINVENT 2.0. This optimization task was parallelized over 10 CPUs and therefore actually corresponded to 1.6 h and 20.2 h respectively. Given access to just 10 CPUs, AHC is able to achieve 200% optimization from the prior in less than a day (21.6 h). This enables optimization tasks to be run on single, local machines (e.g., 6–12 CPUs) on a far more reasonable time scale than previously possible, without the need for cloud computing. This provides opportunities for more than one expensive scoring function (e.g., docking into two receptors, or docking and computer-aided synthesis planning) to be used to evaluate molecule fitness on a more reasonable time scale.

**Table 3:** CPU hours required for RL strategies to optimize the DRD2 docking score benchmark task to different thresholds

	CPU hours required for optimization beyond prior at a given threshold					CPU hours required for optimization beyond external thresholds		
Threshold	120%	140%	160%	180%	200%	Inactive mean	Active mean	80% precision
REINFORCE	74 (0)	173 (0)	– (20)	– (34)	– (96)	2 (0)	103 (0)	177 (0)
REINFORCE + KL regularization	183 (0)	– (0)	– (33)	– (74)	– (216)	22 (0)	204 (0)	– (0)
REINVENT	79 (0)	– (0)	– (8)	– (164)	– (–)	4 (0)	93 (0)	– (0)
REINVENT 2.0	38 (0)	202 (0)	– (16)	– (53)	– (92)	12 (0)	51 (0)	198 (0)
BAR	– (0)	– (0)	– (32)	– (32)	– (–)	4 (0)	0 (0)	– (0)
Hill-Climb	44 (0)	114 (0)	177 (0)	218 (24)	– (85)	16 (0)	57 (0)	99 (0)
Hill-Climb + KL regularization	45 (0)	106 (0)	157 (0)	– (45)	– (45)	8 (0)	58 (0)	99 (0)
Hill-Climb*	11 (0)	31 (1)	52 (6)	– (15)	– (31)	2 (0)	11 (0)	24 (0)
Hill-Climb* + KL regularization	14 (0)	28 (0)	74 (1)	– (17)	– (17)	6 (0)	17 (0)	31 (0)
Augmented Hill-Climb	9 (0)	16 (0)	72 (0)	151 (14)	216 (15)	2 (0)	13 (0)	27 (0)

Time is representative of when the batch mean exceeds the respective internal / external threshold (time of the earliest sample exceeding threshold is shown in brackets). Run using an AMD Threadripper 1920 × CPU and Nvidia GeForce RTX 2060 super GPU. Failing to reach a threshold is marked by a “–”

Additional file 1: Figures S12-17 show the centroids of the largest clusters for the top 100 molecules generated during the six benchmark optimization tasks. Firstly, all strategies are more prone to generating unrealistic chemistry due to the broader training domain of the GuacaMol [22] training set e.g., increasing molecular weight seen in the DRD2 docking score optimization task (Additional file 1: Figure S15). This is even observed for the more heavily regularized REINVENT strategy but is not present when using the MOSES^n^ training set (Fig. 6). Moreover, KL regularization as proposed previously [41, 74] does not seem to improve chemistry generated by REINFORCE and HC and instead shows a tendency to increase molecular weight (Additional file 1: Figure S14). On the other hand, AHC results in chemistry similar to REINVENT and is typically more reasonable than REINVENT 2.0 (e.g., longer linker chains in Additional file 1: Figure S16), is less prone to idiosyncratic tendencies of HC (e.g., large molecules and long chains in Additional file 1: Figure S16)*,* yet more sample-efficient than either. Overall, we believe AHC strikes the right balance in the trade-off between extrapolation and sample-efficiency due to effective, tunable regularization that can maintain training set properties and therefore the generation of sensible and realistic molecules de novo.

### Applying Augmented Hill-Climb to transformer architectures

**Fig. 9:**
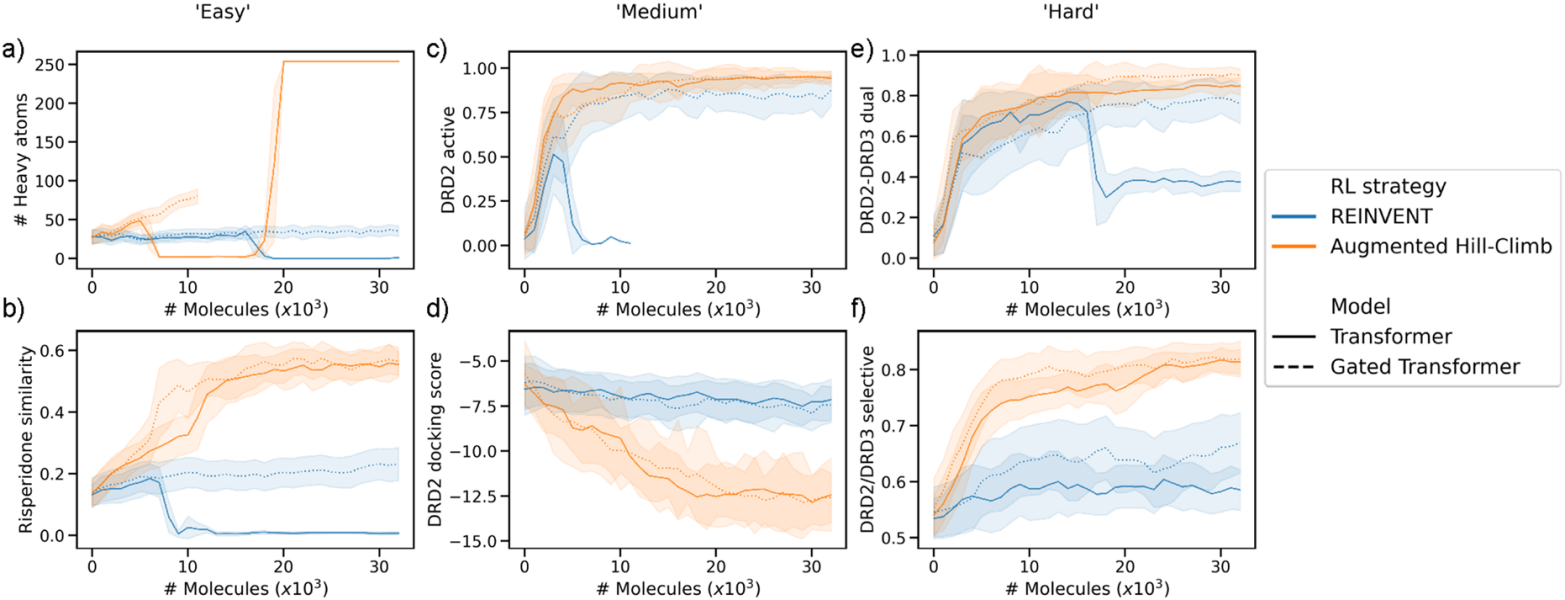
Per-molecule optimization by REINVENT and Augmented Hill-Climb RL strategies for the transformer (Tr) and gated transformer (GTr) architecture against the DRD2 benchmark objectives. Tr is more unstable during RL by REINVENT which is stabilized by the GTr. In all cases Augmented Hill-Climb outperforms REINVENT at objective optimization. Although these transformer models are more prone to mode collapse than an RNN as observed by a drop in validity and uniqueness as shown in Additional file 1: Figures S18-19

RL algorithms (including AHC) should be model-agnostic and therefore applicable to other models used in a policy-based reinforcement learning setting. To test this and confirm whether AHC is still superior to REINVENT in this setting, we applied these two RL approaches to a transformer model (Tr) that uses state-of-the-art attention mechanisms [68] (Experiment 5, Fig. 1). Rather than typical seq-to-seq prediction, the encoder was trained in an autoregressive manner to predict the next token in a SMILES sequence by attending over all previous tokens. RL was then conducted using the same approach as with the RNN on the same DRD2-based benchmark applied previously, for both REINVENT and AHC in combination with DF3 (a more stringent diversity filter that penalizes all molecules independent of score). Figure 9 shows that AHC still outperforms REINVENT with regards to sample-efficiency and optimization power. However, as shown in Fig. 9a–c, and e the Tr model is much less stable under RL optimization compared to the RNN and more readily undergoes mode collapse i.e., it starts generating invalid or repeated (non-unique) molecules, as shown in Additional file 1: Figures S18-19. In fact, very few implementations of transformers exist within a RL setting (e.g., [104, 105]) likely due to this instability during training and computational expense, sometimes being distilled to an RNN for RL [10]. Therefore we additionally implemented a modified transformer architecture designed to stabilize model optimization during RL [69]. This gated transformer (GTr) architecture implements a GRU-like gate in-place of the residual connection and relocates layer normalization to input streams (notably this is not the only recent example of combining concepts from GRUs or LSTMs with transformer architectures [106]). As shown in Fig. 9, this appeared to stabilize RL and again showed that AHC outperforms REINVENT, leaving only the heavy atom task still failing with AHC which is notably outside the applicability domain of the training dataset (and also devoid of any practical relevance). Examples of de novo chemistry generated by these models can be seen in Additional file 1: Figures S20-25. Overall, this shows that RL efficiency gains by AHC also generalize to other language models.

### Perspective

Many more complex architectures to conduct de novo molecular design have been published since seminal works [3, 4, 12] however there has been little convincing evidence of any significant improvement over RNNs since that time. In fact, REINVENT recently displayed state-of-the-art performance on molecular optimization within a given number of samples [107]. Therefore, we believe that AHC currently evidences state-of-the-art goal-directed de novo molecule generation due to superior performance over REINVENT. That said we also underline that progress still needs to be made in how we evaluate and compare state-of-the-art, not only with regards to scoring function optimization but also the chemistry generated.

We acknowledge that alternative methods can be used to improve the sample-efficiency of RL [108]. For example, experience replay can be used to remind the agent of ‘good’ molecules [58, 108], a margin guard [109] can be employed to dynamically change α during RL updates or curriculum learning can be used to accelerate learning by breaking the objective into a sequence of simpler tasks [110]. We are of the opinion that AHC is a more direct and principled approach to improve sample-efficiency and could even be used in combination with these methods to potentially further improve reinforcement learning for de novo molecule optimization.

## Conclusion

In this work, we have proposed a modification to the REINVENT [4, 58] RL framework for language-based de novo molecule generation that exhibits improved sample-efficiency. This method, referred to as Augmented Hill-Climb, improves optimization ability ~ 1.5-fold over REINVENT for the task of optimizing DRD2 Glide-SP [89] docking score. While more susceptible to mode collapse, this can be successfully ameliorated by application of an appropriate diversity filter. This new strategy can optimize the docking score for other systems beyond DRD2 including OPRM1, AGTR1 and OX1R where it improved sample-efficiency ~ 45-fold on average. When compared to other common RL strategies used in language-based RNN de novo molecule generation [5, 22, 41], it was found to outperform REINFORCE*,* REINVENT*,* BAR and Hill-Climb with respect to optimization ability, sample-efficiency, regularization and resulted in chemically reasonable molecules. We believe this is achieved by circumventing unwarranted regularization in REINVENT, but it can also be viewed as applying essential regularization to Hill-Climb. Furthermore, we show that this algorithm can be successfully applied to transformer architectures showing that it generalizes across language models. The improvement in sample-efficiency enabled by Augmented Hill-Climb will be especially useful when using computationally expensive scoring functions such as molecular docking or computer-aided synthesis planning tools. We believe these results highlight there is still scope for improvement in early generation ML-based generative models and that designing more complex generative models is not the only path to advance the field of molecular de novo design.

## Supplementary Information

**Additional file 1. **Supplementary tables and figures.

## Data Availability

The datasets supporting the conclusions of this article can be found via the following link https://doi.org/10.6084/m9.figshare.19591024.v1. The generative model code and scoring function code used to obtain the results discussed within the article is available at https://github.com/MorganCThomas/SMILES-RNN and https://github.com/MorganCThomas/MolScore, respectively.

## Abbreviations

ADAM: Adaptative moment estimation
AGTR1: AT_1_ receptor
AHC: Augmented Hill-Climb
AI: Artificial Intelligence
BAR: Best agent reminder
DF: Diversity filter
DRD2: D_2_ receptor D2
GPCR: G protein-coupled receptor
ECFP: Extended-connectivity fingerprint
GRU: Gated recurrent unit
HC: Hill-Climb
KL: Kullback–Leibler
LSTM: Long short-term memory
ML: Machine learning
MPO: Multi-parameter optimization
OPRM1: µ Receptor
OX1R: OX_1_ receptor
QSAR: Quantitative structure–activity relationship
RL: Reinforcement learning
RNN: Recurrent neural network
SMILES: Simplified molecular-input line-entry system
Tr: Transformer
GTr: Gated transformer
