# Supplementary material for: Augmented Hill-Climb increases reinforcement learning efficiency for language-based de novo molecule generation
Source: J Cheminform. 2022 Oct 3;14:68. doi: 10.1186/s13321-022-00646-z (PMC9531503; doi:10.1186/s13321-022-00646-z)
Supplement: Supplementary file 1 — Additional file 1. Supplementary tables and figures. [file 13321_2022_646_MOESM1_ESM.pdf]

Supporting Information for

Augmented Hill-Climb increases reinforcement learning efficiency for language-based *de novo* molecule generation

Morgan Thomas[1], Noel M. O'Boyle[2], Andreas Bender[1*] and Chris de Graaf[2*]

[1] Centre for Molecular Informatics, Department of Chemistry, University of Cambridge, Cambridge, CB2 1EW, UK

[2] Computational Chemistry, Sosei Heptares, Steinmetz Building, Granta Park, Great Abington, Cambridge, CB21 6DG, UK

E-mail: ab454@cam.ac.uk, chris.degraaf@soseiheptares.com

Supplementary Tables

Table S1: Intrinsic metrics of 10,000 sampled *de novo* molecules from the recurrent neural network (RNN), Transformer (Tr) and Gated Transformer (GTr) used in this work when trained on the GuacaMol training dataset. These metrics include the ratio of valid, unique, scaffold unique and novel molecules, as well as sphere exclusion diverse (ECFP4 of length 1,204 with sphere radius equal to Tanimoto distance of 0.65), functional group diverse (FG) and ring system diverse (RS). Lastly, the ratio of molecules passing MOSES filters.

**Model**	**Valid** (↑)	**Unique** (↑)	**Novel** (↑)	**SEDiv@1k** (↑)	**Scaffold Unique** (↑)	**FG** (↑)	**RS** (↑)	**MOSES Filters** (↑)
RNN	0.96	0.99	0.96	0.88	0.84	0.20	0.18	0.52
Tr	0.96	1.00	0.97	0.85	0.86	0.18	0.17	0.52
GTr	0.94	0.99	0.97	0.89	0.87	0.21	0.18	0.50

Table S2: Extrinsic metrics of 10,000 sampled *de novo* molecules from the recurrent neural network (RNN), Transformer (Tr) and Gated Transformer (GTr) used in this work when trained on the GuacaMol training dataset. These metrics measure the similarity of *de novo* molecules to the GuacaMol test dataset. Including the analogue similarity (ratio of *de novo* molecules that have test set analogues), analogue coverage (ratio of test set with *de novo* analogues), average single nearest neighbour similarity (SNN), cosine similarity of functional groups (FG) and ring systems (RS), fragments (Frag) and scaffolds (Scaff), and Wasserstein distance of LogP, natural product likeness (NP), synthetic accessibility (SA), quantitative estimate of drug-likeness (QED) and molecular weight distributions. Note that fingerprints similarities are based on ECFP4 fingerprints of length 1,024 and analogues are defined as having a Tanimoto similarity greater than 0.4.

**Model**	**Analogue Similarity** (↑)	**Analogue Coverage** (↑)	**SNN** (↑)	**FG** (↑)	**RS** (↑)	**Frag** (↑)	**Scaff** (↑)	**logP** (↓)	**NP** (↓)	**SA** (↓)	**QED** (↓)	**Weight** (↓)
RNN	0.83	0.43	0.52	1.00	1.00	1.00	0.64	0.02	0.04	0.08	0.01	13.01
Tr	0.82	0.40	0.51	1.00	1.00	1.00	0.59	0.07	0.08	0.04	0.01	2.90
GTr	0.76	0.36	0.49	1.00	1.00	1.00	0.55	0.07	0.07	0.03	0.00	3.47

Table S3: Default hyperparameters used for reinforcement learning strategies benchmarked in this work. Additional configurations with regularization and disclosed in square brackets.

**RL strategy**	**Training steps**	**Batch size**	σ	k	λ_{KL}	lr	α
REINFORCE [+reg]	500	64	-	-	[10]	1×10^{-4}	-
REINVENT	500	64	60	-	-	5×10^{-4}	-
REINVENT 2.0	250	128	120	-	-	5×10^{-4}	-
BAR	500	64	60	-	-	5×10^{-4}	0.5
Augmented Hill-Climb	500	64	60	50%	-	5×10^{-4}	-
Hill-Climb [+reg]	32	1024	-	50%	[10]	5×10^{-4}	-
Hill-Climb* [+reg]	500	64	-	50%	[10]	5×10^{-4}	-

Table S4: Diversity filter configurations used in this work.

	**DF1**	**DF2**	**DF3**
Topology simplification	BM scaffold	BM scaffold	BM scaffold
Fingerprint type	ECFP (Morgan)	ECFP (Morgan)	ECFP (Morgan)
Fingerprint radius	2	2	2
Fingerprint bits	1024	1024	1024
Similarity metric	Tanimoto	Tanimoto	Tanimoto
Minimum score threshold	0.8	0.5	0.0
Bin size	25	50	50
Output mode	Binary	Linear	Linear

Table S5: Number of molecules downloaded from ExCAPE-DB and those docked against targets used in this work to assess retrospective performance.

	**ExCAPE-DB**		**Docked**	
	Active	Inactive	Active	Inactive
DRD2	4613	343076	3734	9538
OPRM1	3128	2786	3125	2573
AGTR1	671	558	270	516
OX1R	681	322795	564	9860

Supplementary Figures

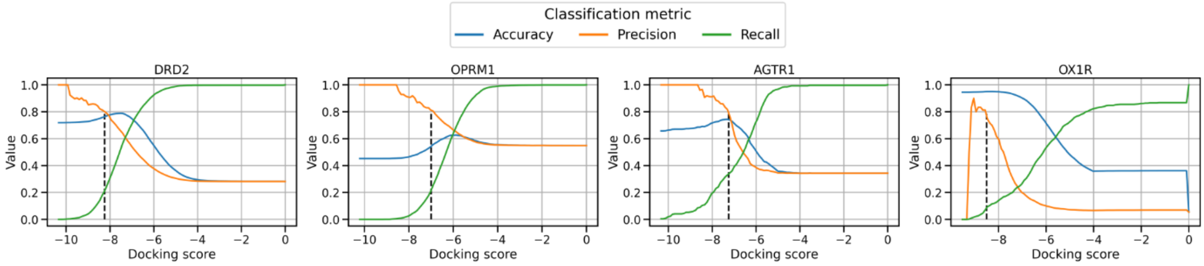

Figure S1: Retrospective classification performance of docking protocol on the four targets investigated in this work. In each case, the accuracy, precision and recall are traced with varying docking score decision thresholds. Thresholds corresponding to ~ 80% precision are annotated by black dashed lines.

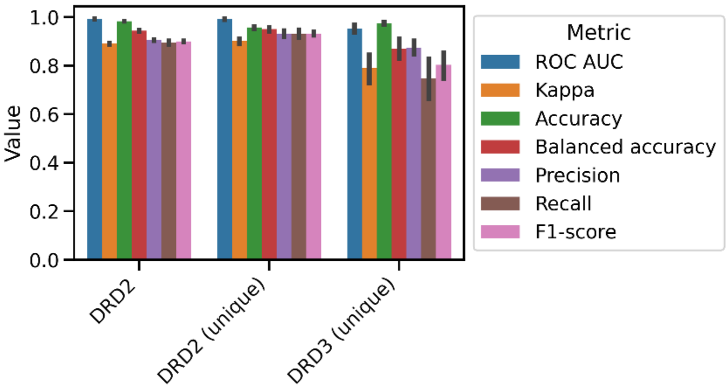

Figure S2: QSAR model performance of RF models trained on DRD2 and DRD3 active and inactive molecules, based on 5-fold stratified, clustered cross-validation with GHOST [85] decision threshold identification.

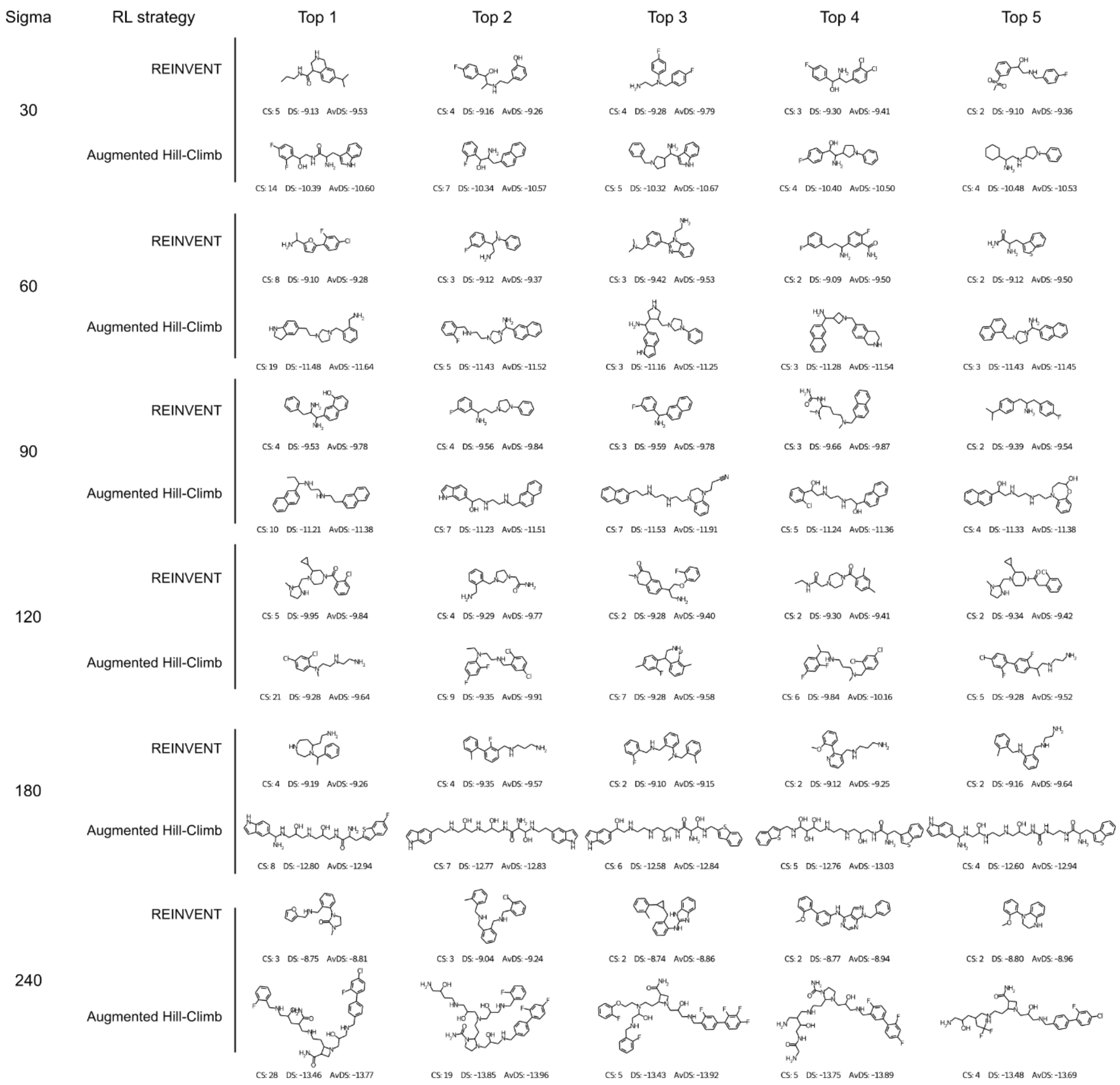

Figure S3: Centroid of the top 5 largest clusters for the top 100 molecules generated by REINVENT and Augmented Hill-Climb (both with DF1) according to docking score against DRD2 receptor for varying values of sigma (σ). Cluster size (CS), centroid docking score (DS) and the average cluster docking score (AvDS) is annotated below.

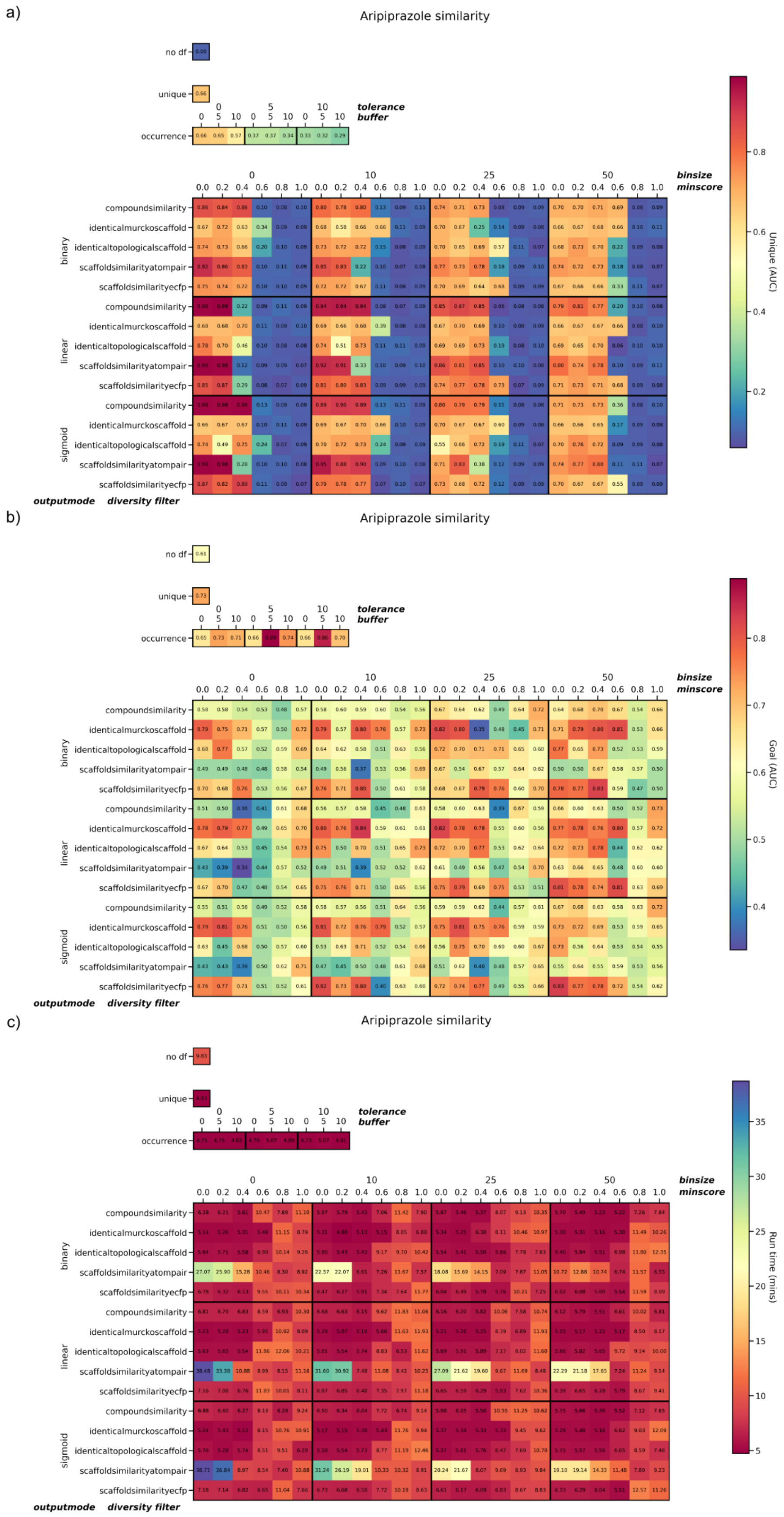

Figure S4: Optimization of Aripiprazole similarity task with different diversity filters and their parameters. Three endpoints are measured: (**a**) unique area under the curve (AUC), (**b**) goal AUC (**c**) and run time.

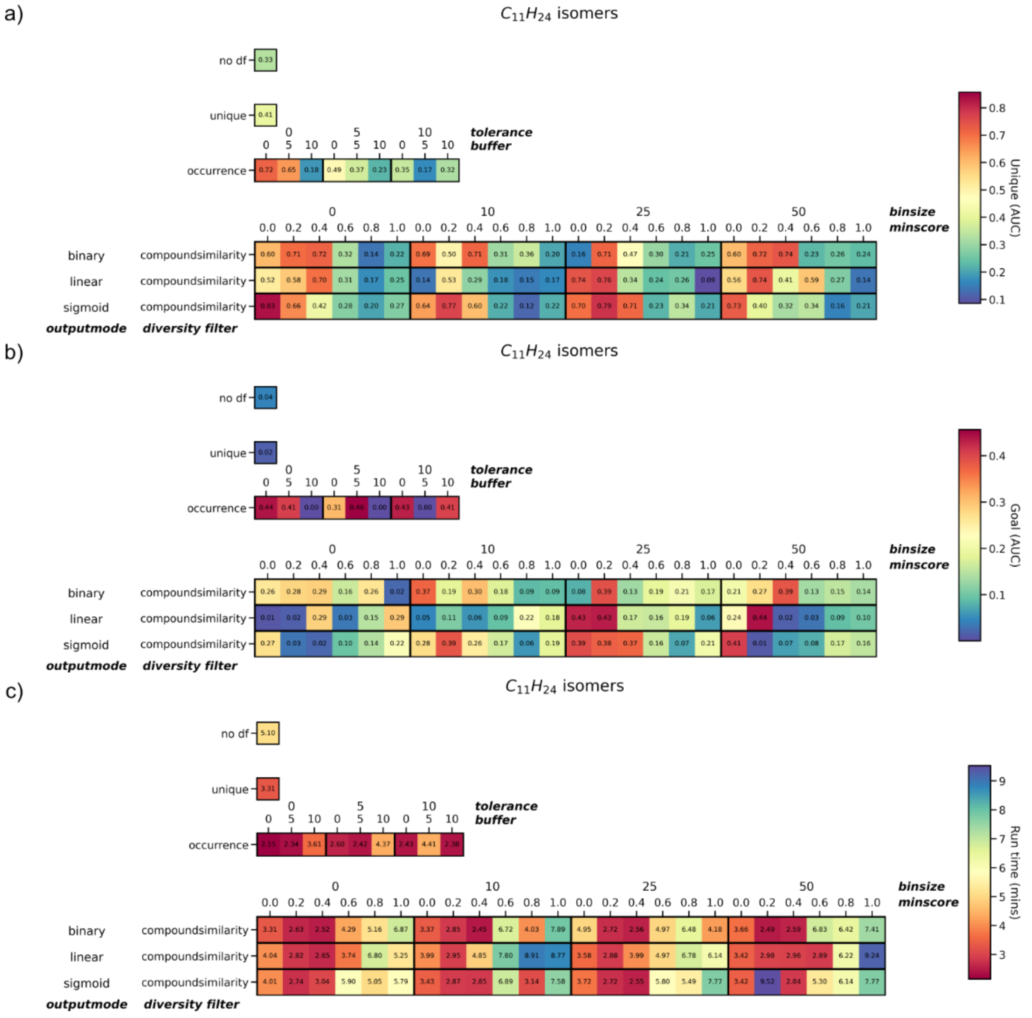

Figure S5: Optimization of $C_{11}H_{24}$ isomers task with different diversity filters and their parameters. Three endpoints are measured: (**a**) unique area under the curve (AUC), (**b**) goal AUC (**c**) and run time. Note: scaffold-based diversity filters aren't shown as $C_{11}H_{24}$ isomers cannot form rings and satisfy the molecular formula and so no scaffolds will be detected.

a)

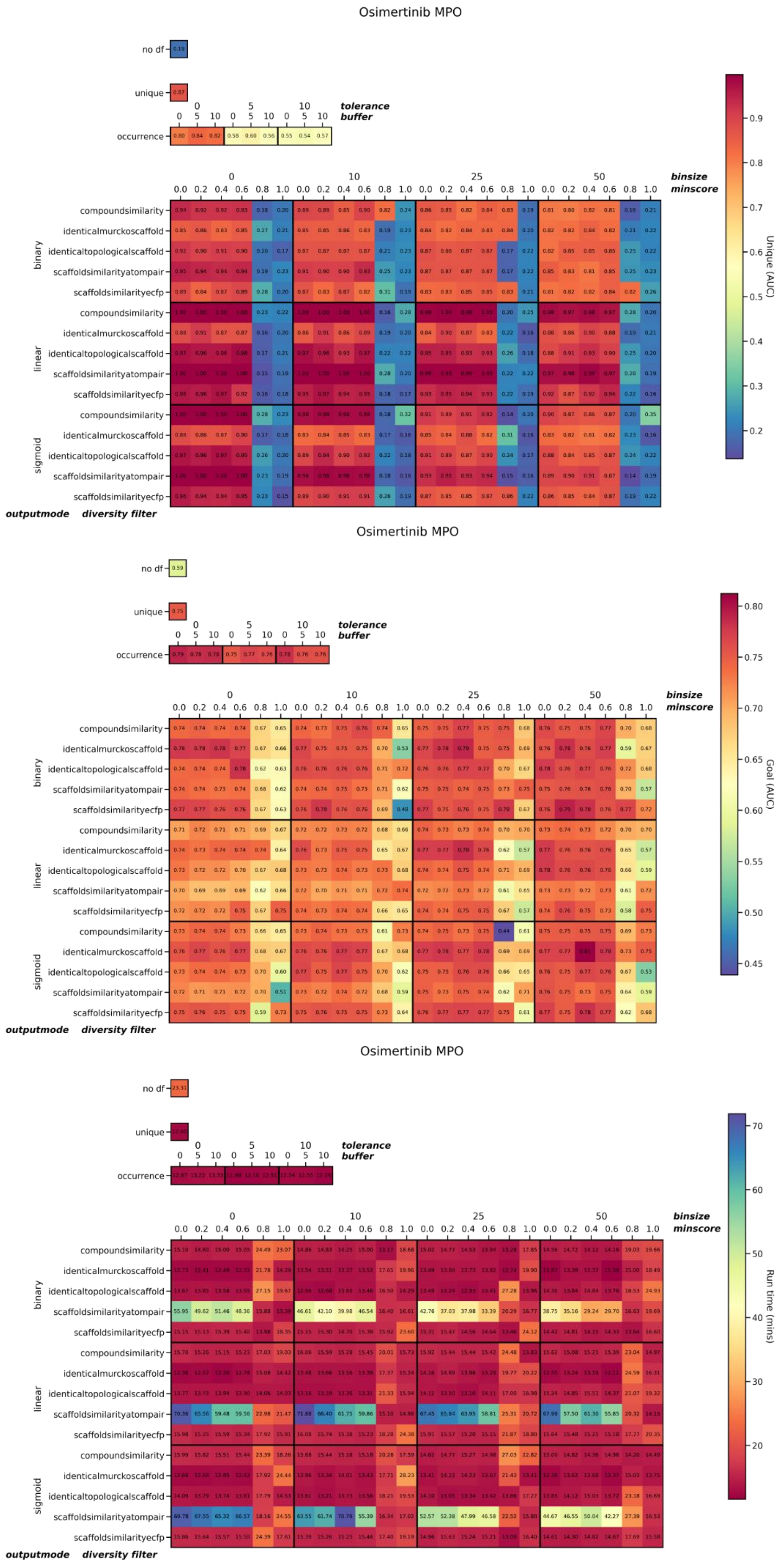

Figure S6: Optimization of Osimertinib MPO task with different diversity filters and their parameters. Three endpoints are measured: (**a**) unique area under the curve (AUC), (**b**) goal AUC (**c**) and run time.

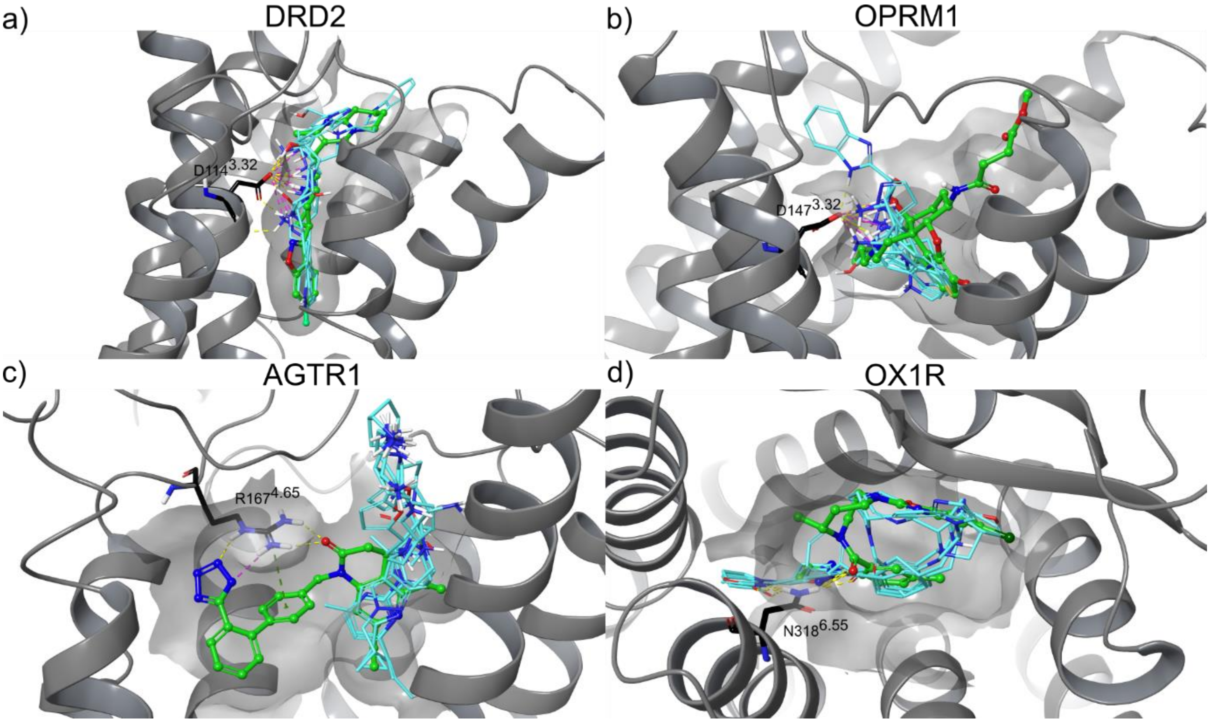

Figure S7: Docked poses of the centroid molecules (cyan) shown in Figure 5 generated by AHC + DF2 compared to the co-crystallized ligand (green) for each respective target. For (a) DRD2 and (b) OPRM1, RL and docking results in sensible pose generation satisfying crucial residue interactions with D114^{3x32}. While for (c) AGTR1 poses occupy only one relevant sub-pocket compared to the co-crystal ligand and form no interactions with R167^{4x65}. Only one pose for (d) OX1R mimics the horseshoe shape adopted by the co-crystal ligand, however, most form an interaction with N318^{6x55}.

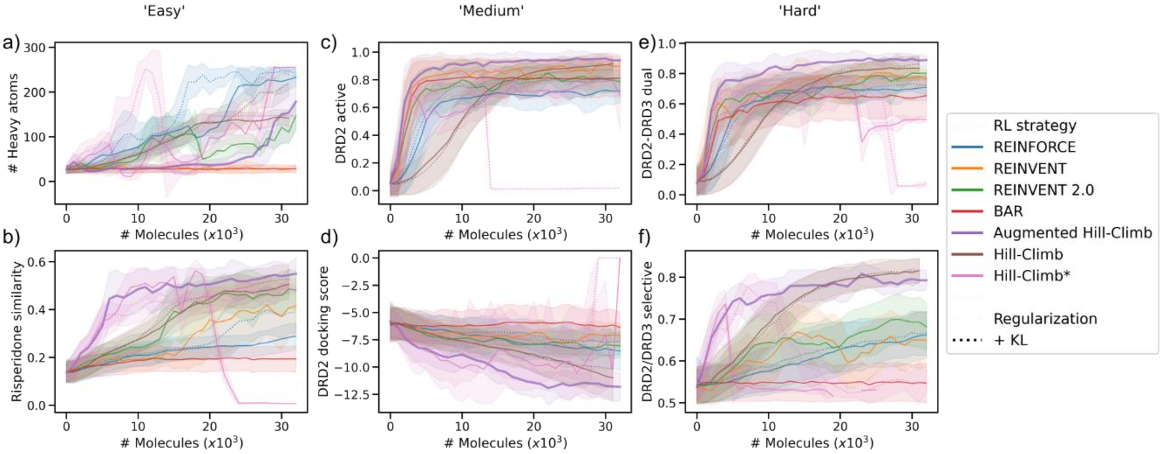

Figure S8: Per-molecule optimization for objective optimization using different RL strategies. In all cases, except the number of heavy atoms, AHC outperforms all other RL strategies with respect to objective optimization while maintaining validity and uniqueness. Only valid molecules are plotted, therefore gaps seen with HC* denote regions where no valid molecules were generated.

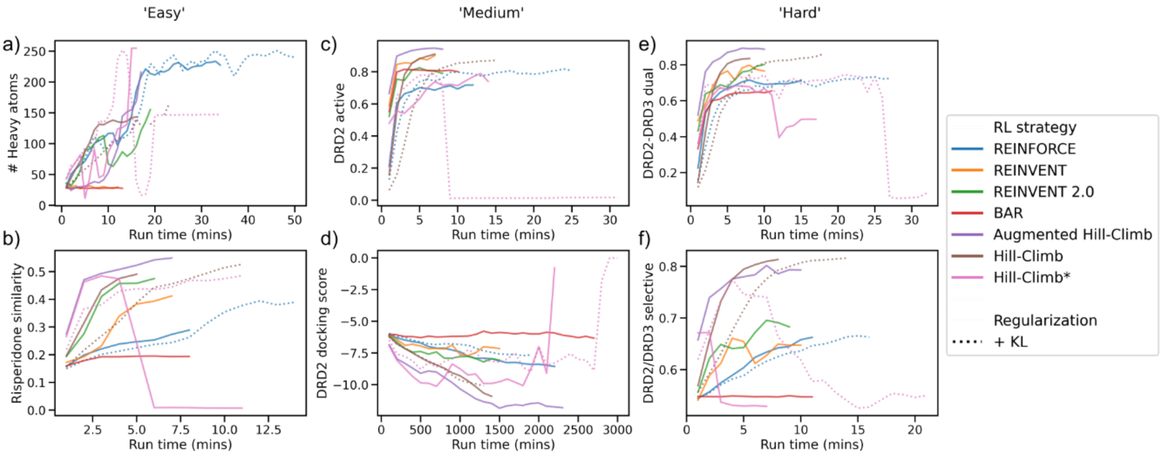

Figure S9: Wall time for objective optimization using different RL strategies. Run using an AMD Threadripper 1920x CPU and Nvidia GeForce RTX 2060 super GPU. The docking tasks were parallelized over 10 CPU cores while all other tasks used only 1 CPU core.

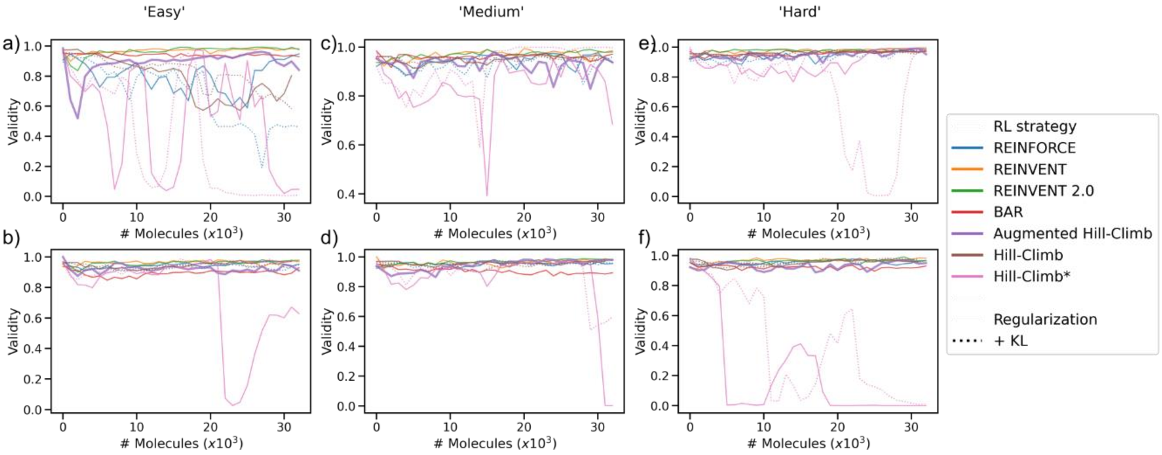

Figure S10: Validity for objective optimization using different RL strategies. HC* suffers from a drop in validity.

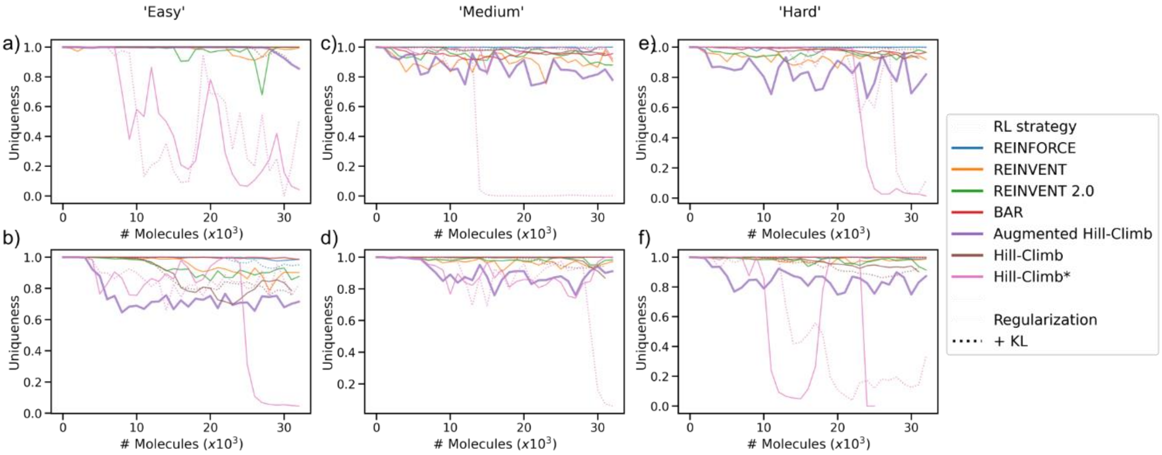

Figure S11: Uniqueness for objective optimization using different RL strategies. HC* suffers from a drop in uniqueness.

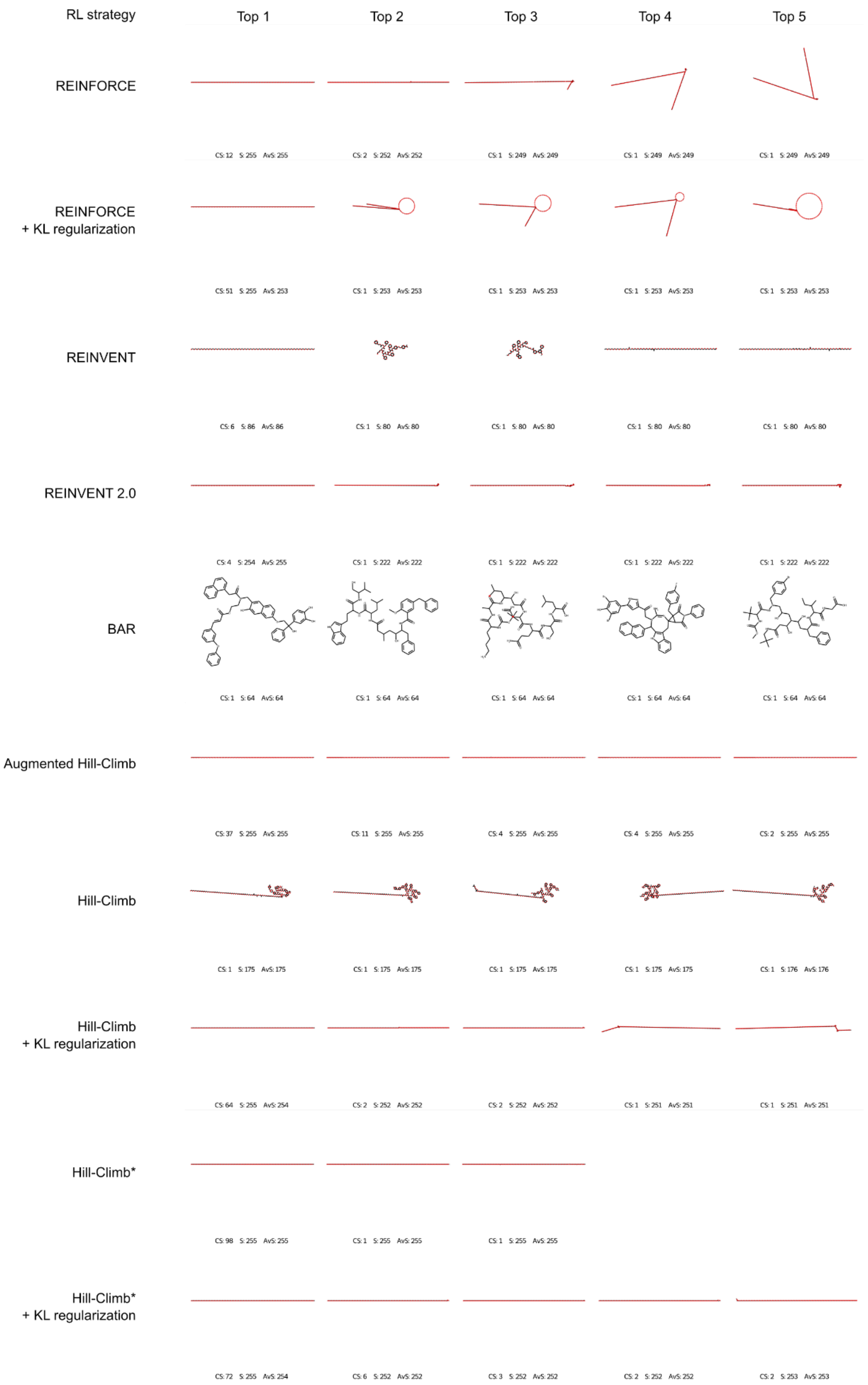

Figure S12: Centroid of the top 5 largest clusters for the top 100 molecules in the heavy atom task for different RL strategies. Cluster size (CS), centroid score (S) and the average cluster score (AvS) is annotated below.

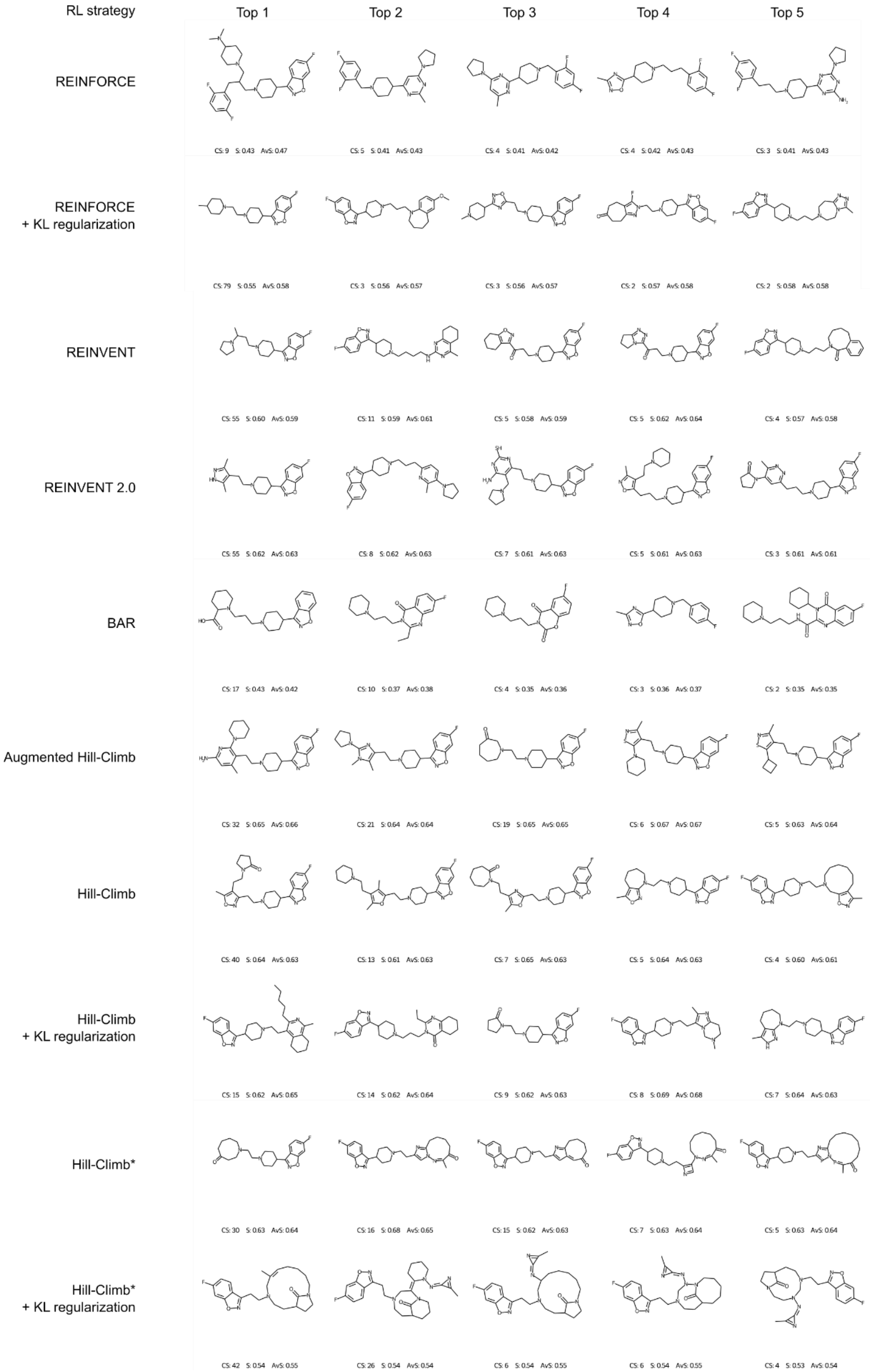

Figure S13: Centroid of the top 5 largest clusters for the top 100 molecules in the Risperidone similarity task for different RL strategies. Cluster size (CS), centroid score (S) and the average cluster score (AvS) is annotated below.

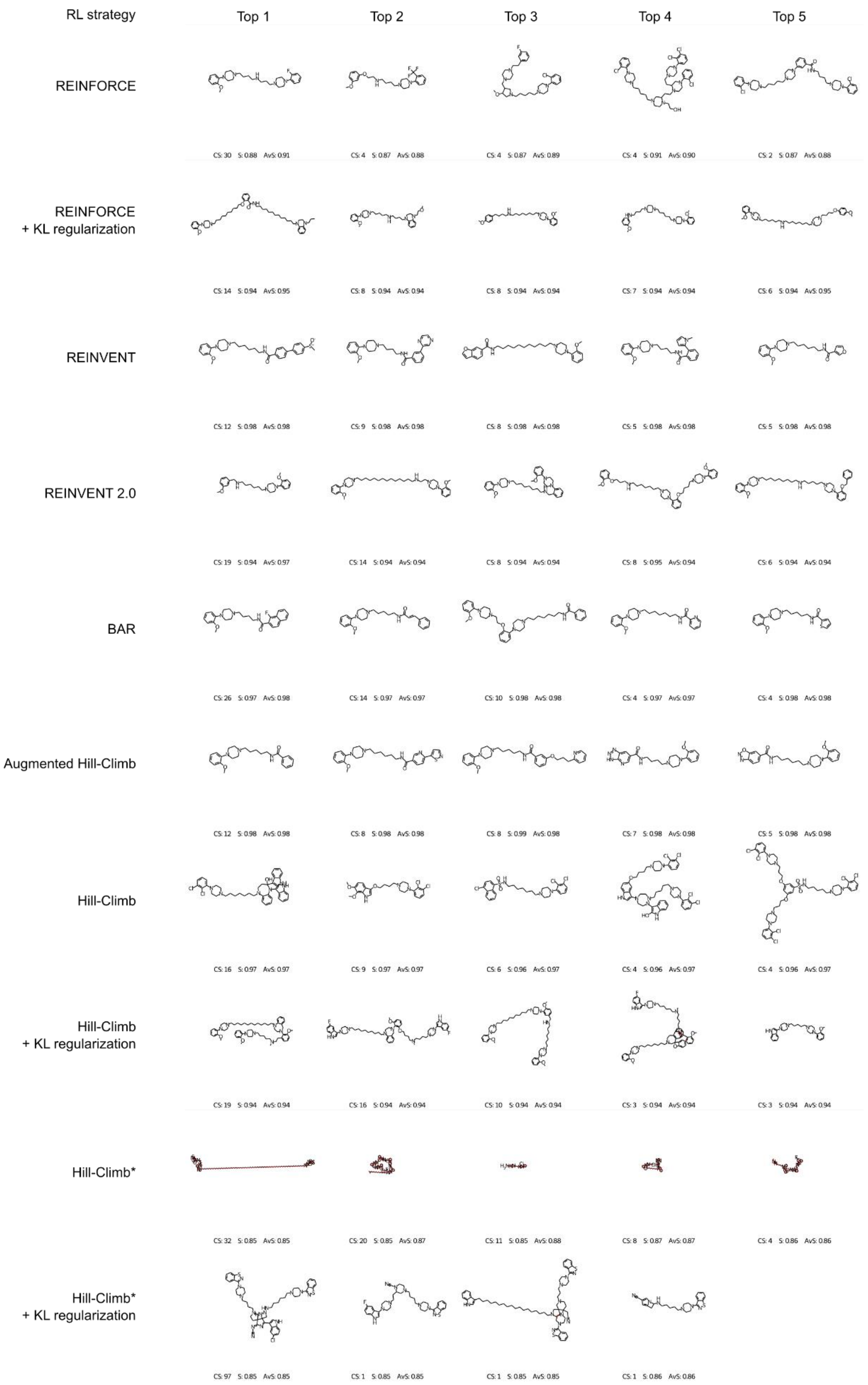

Figure S14: Centroid of the top 5 largest clusters for the top 100 molecules in the DRD2 activity task for different RL strategies. Cluster size (CS), centroid score (S) and the average cluster score (AvS) is annotated below.

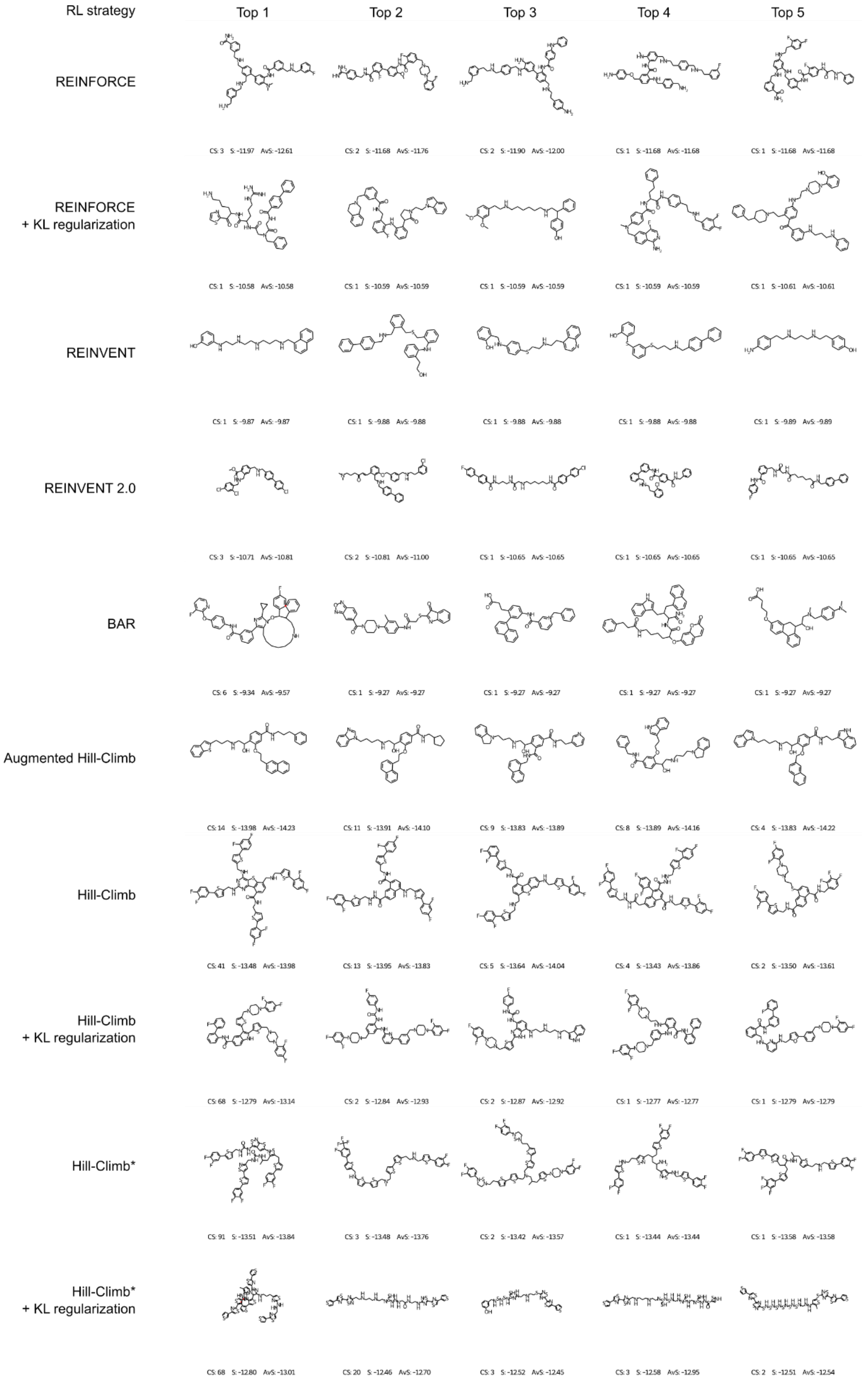

Figure S15: Centroid of the top 5 largest clusters for the top 100 molecules in the DRD2 docking score task for different RL strategies. Cluster size (CS), centroid score (S) and the average cluster score (AvS) is annotated below.

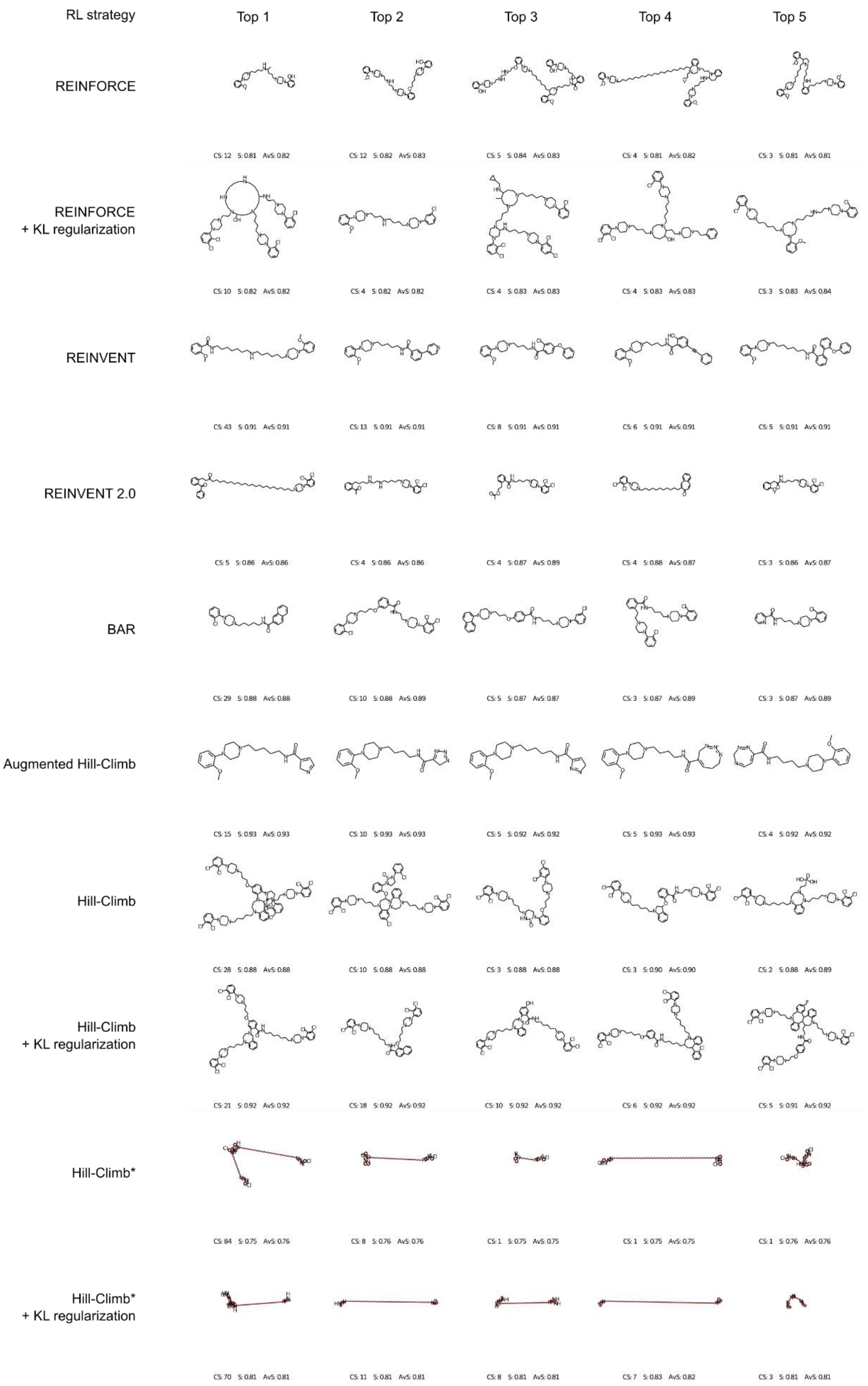

Figure S16: Centroid of the top 5 largest clusters for the top 100 molecules in the DRD2-DRD3 QSAR dual predicted probability of activity task for different RL strategies. Cluster size (CS), centroid score (S) and the average cluster score (AvS) is annotated below. Note where AHC generates cationic species due to imperfections in the neutralization of training data resulting in charge symbols in the RNN vocabulary.

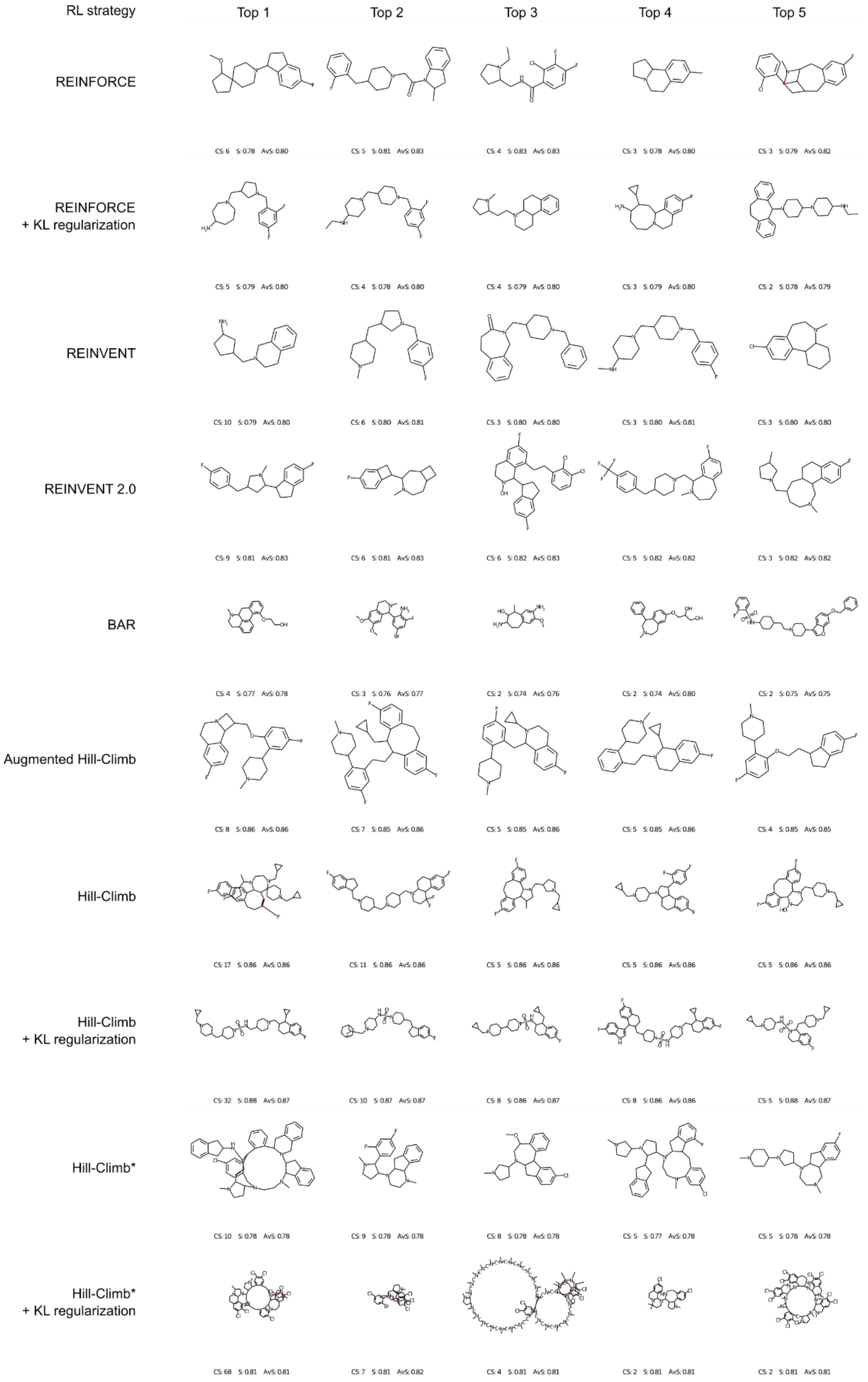

Figure S17: Centroid of the top 5 largest clusters for the top 100 molecules in the DRD2/DRD3 QSAR predicted probability of selective activity task for different RL strategies. Cluster size (CS), centroid score (S) and the average cluster score (AvS) is annotated below.

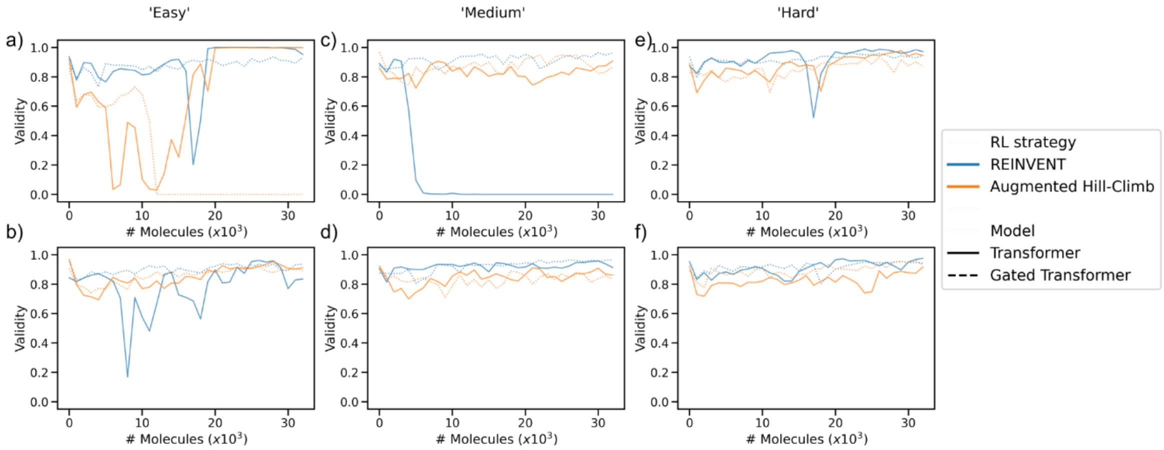

Figure S18: Validity for objective optimization using REINVENT and Augmented Hill-Climb with a transformer or gated transformer model.

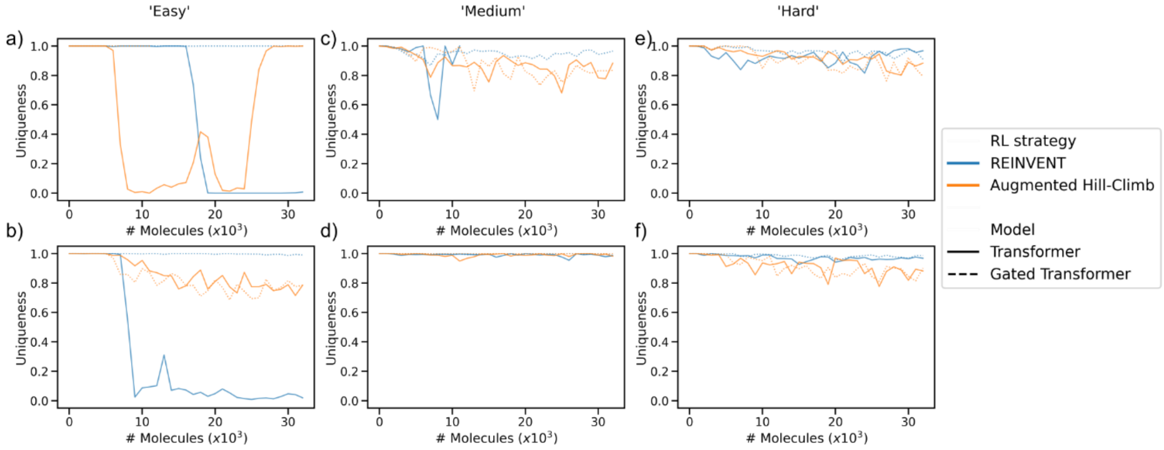

Figure S19: Uniqueness for objective optimization using REINVENT and Augmented Hill-Climb with a transformer or gated transformer model. Transformer model is more prone to undergoing a drop in uniqueness.

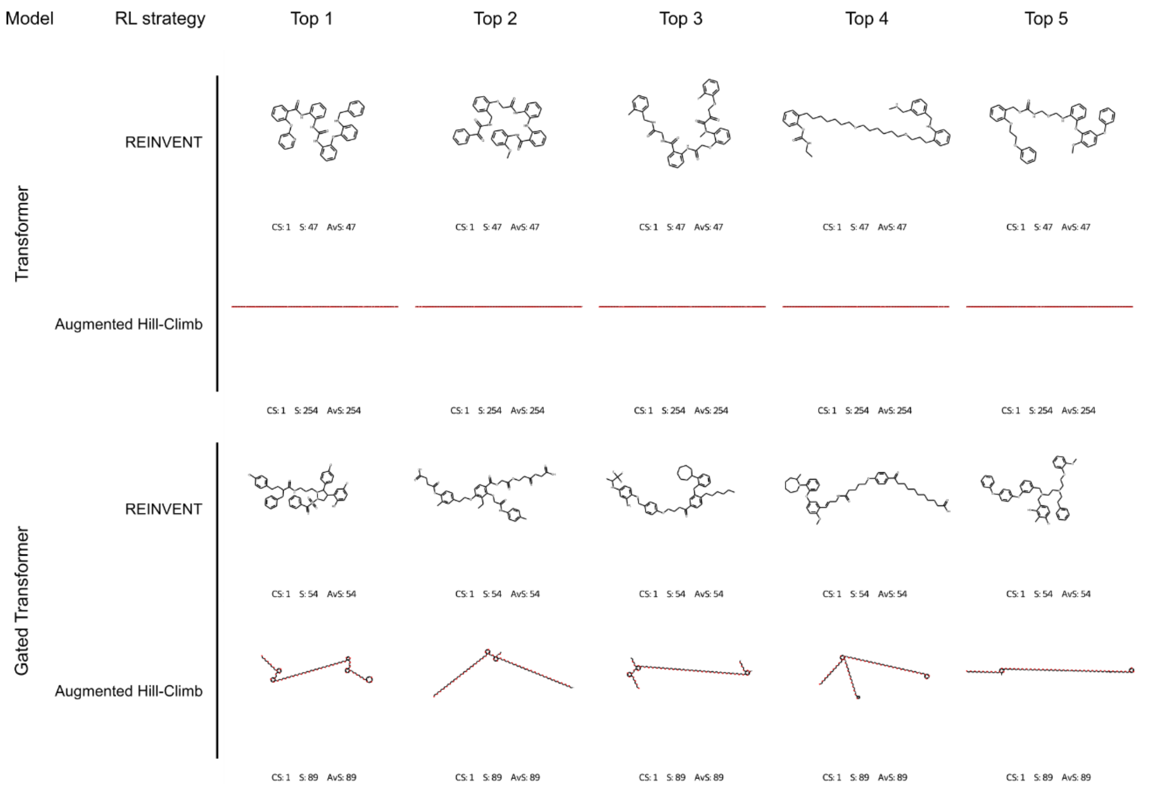

Figure S20: Centroid of the top 5 largest clusters for the top 100 molecules in the heavy atom task for transformer models. Cluster size (CS), centroid score (S) and the average cluster score (AvS) is annotated below.

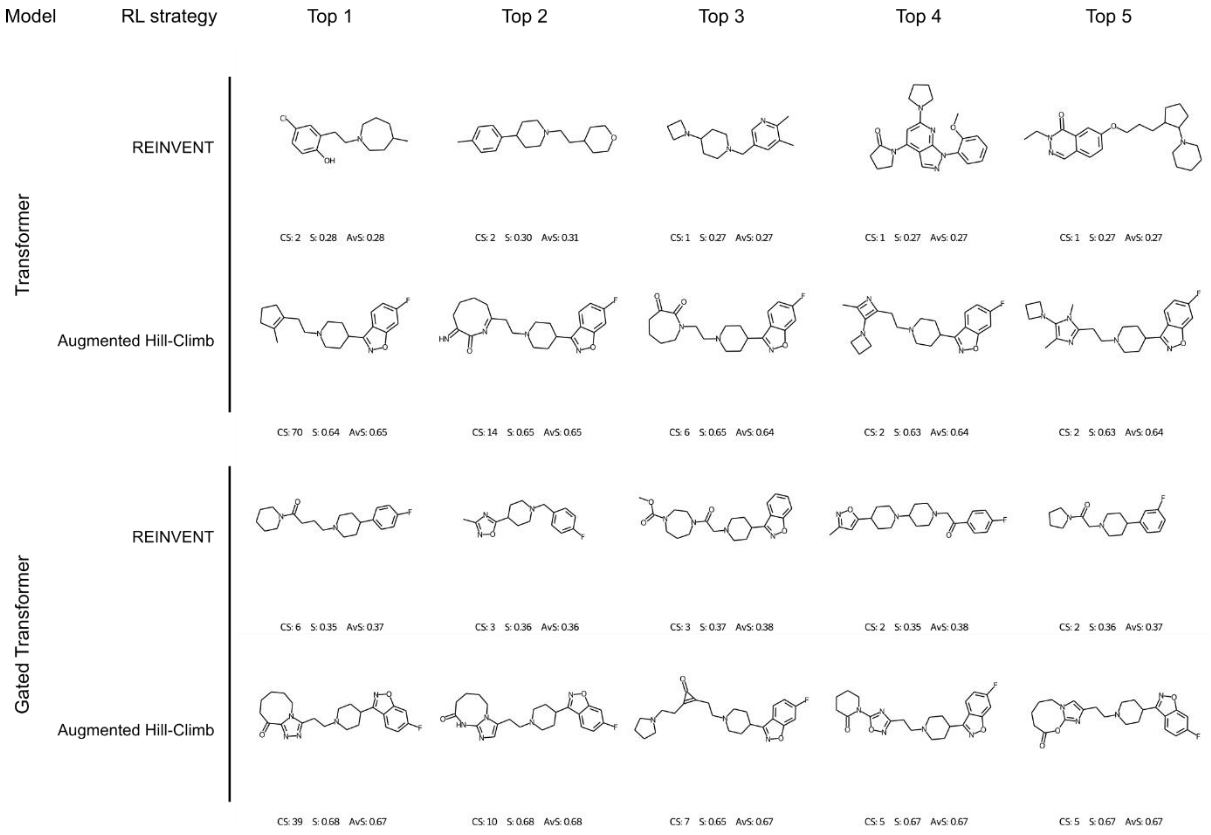

Figure S21: Centroid of the top 5 largest clusters for the top 100 molecules in the Risperidone similarity task for transformer models. Cluster size (CS), centroid score (S) and the average cluster score (AvS) is annotated below.

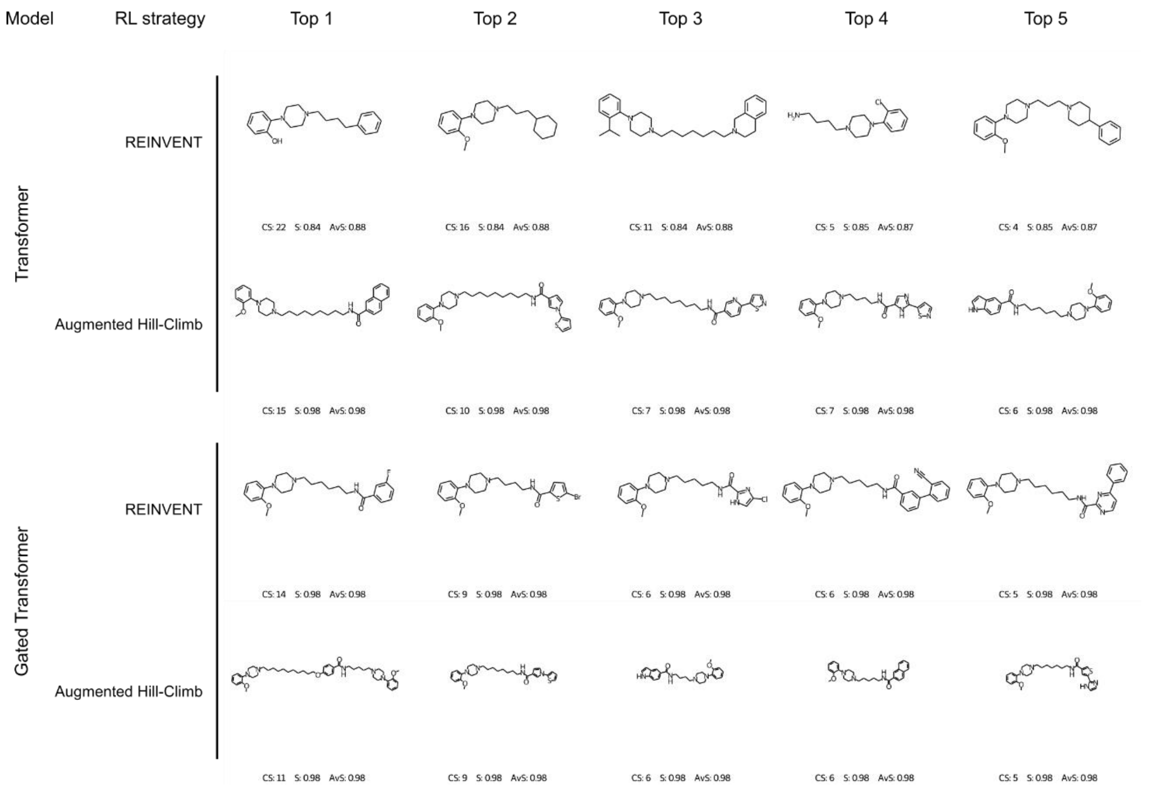

Figure S22: Centroid of the top 5 largest clusters for the top 100 molecules in the DRD2 activity task for transformer models. Cluster size (CS), centroid score (S) and the average cluster score (AvS) is annotated below.

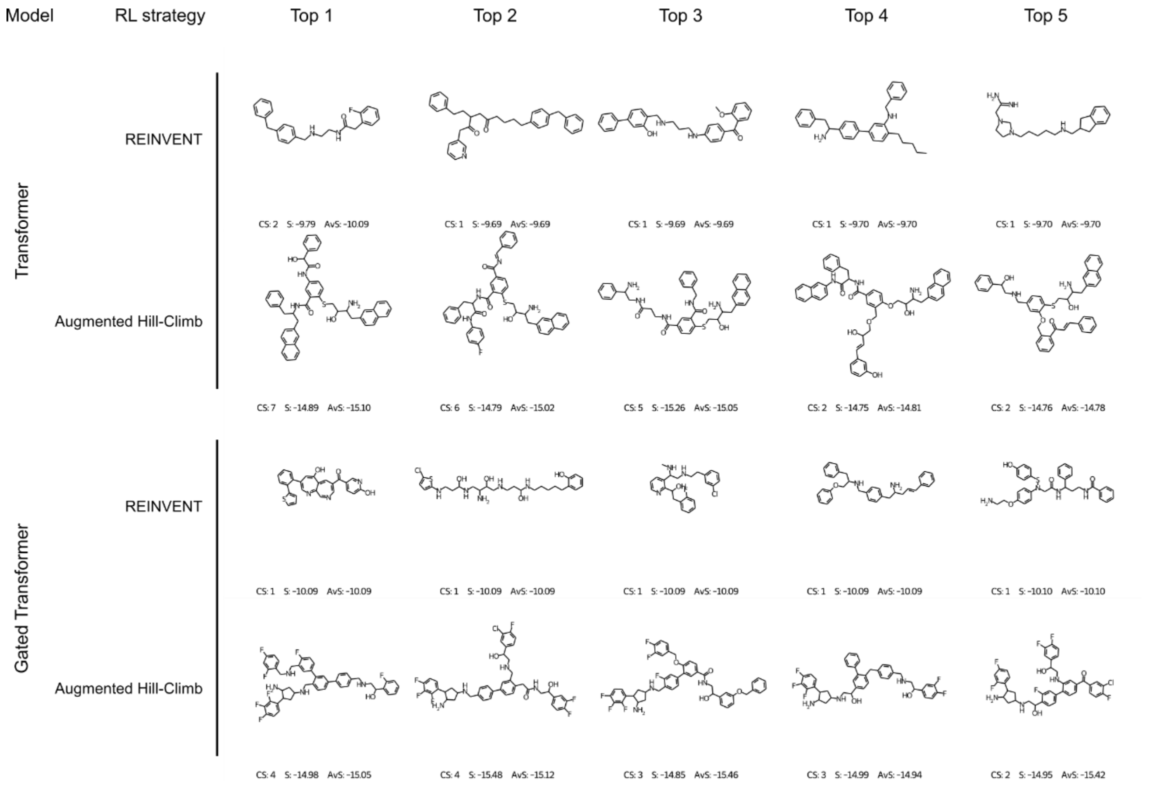

Figure S23: Centroid of the top 5 largest clusters for the top 100 molecules in the DRD2 docking score task for transformer models. Cluster size (CS), centroid score (S) and the average cluster score (AvS) is annotated below.

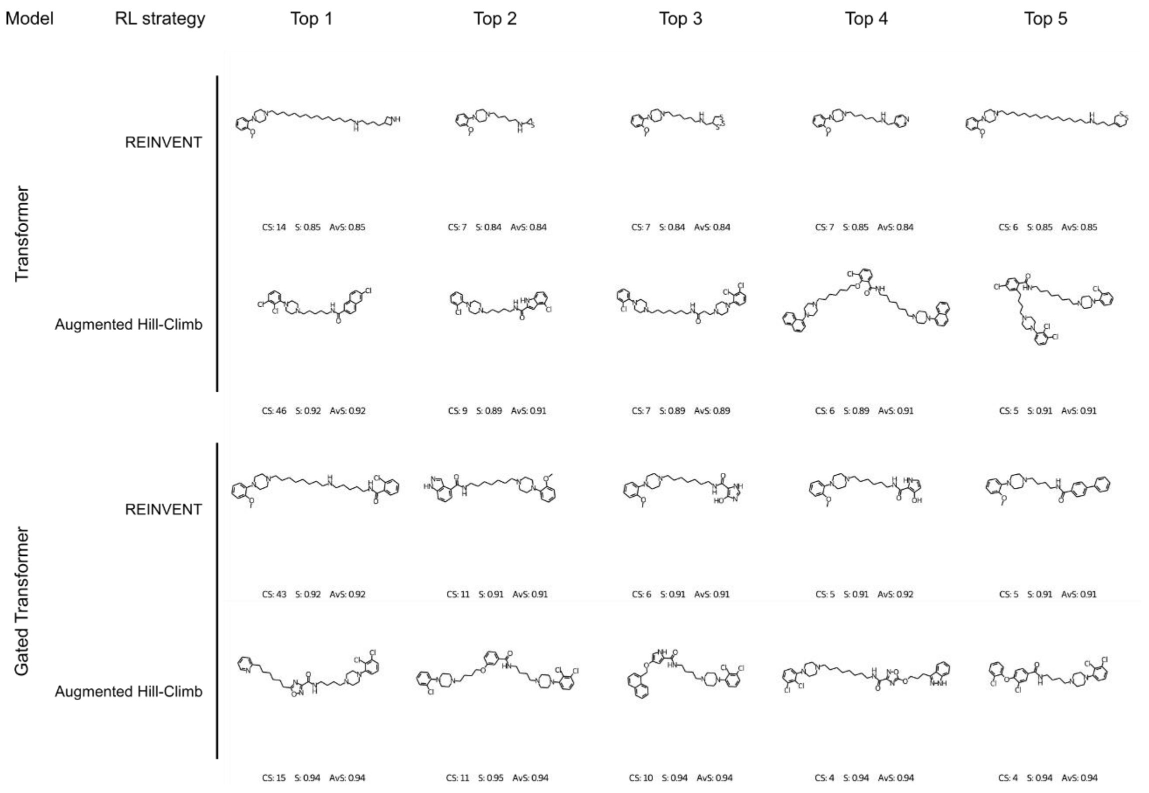

Figure S24: Centroid of the top 5 largest clusters for the top 100 molecules in the DRD2-DRD3 QSAR dual predicted probability of activity task for transformer models. Cluster size (CS), centroid score (S) and the average cluster score (AvS) is annotated below.

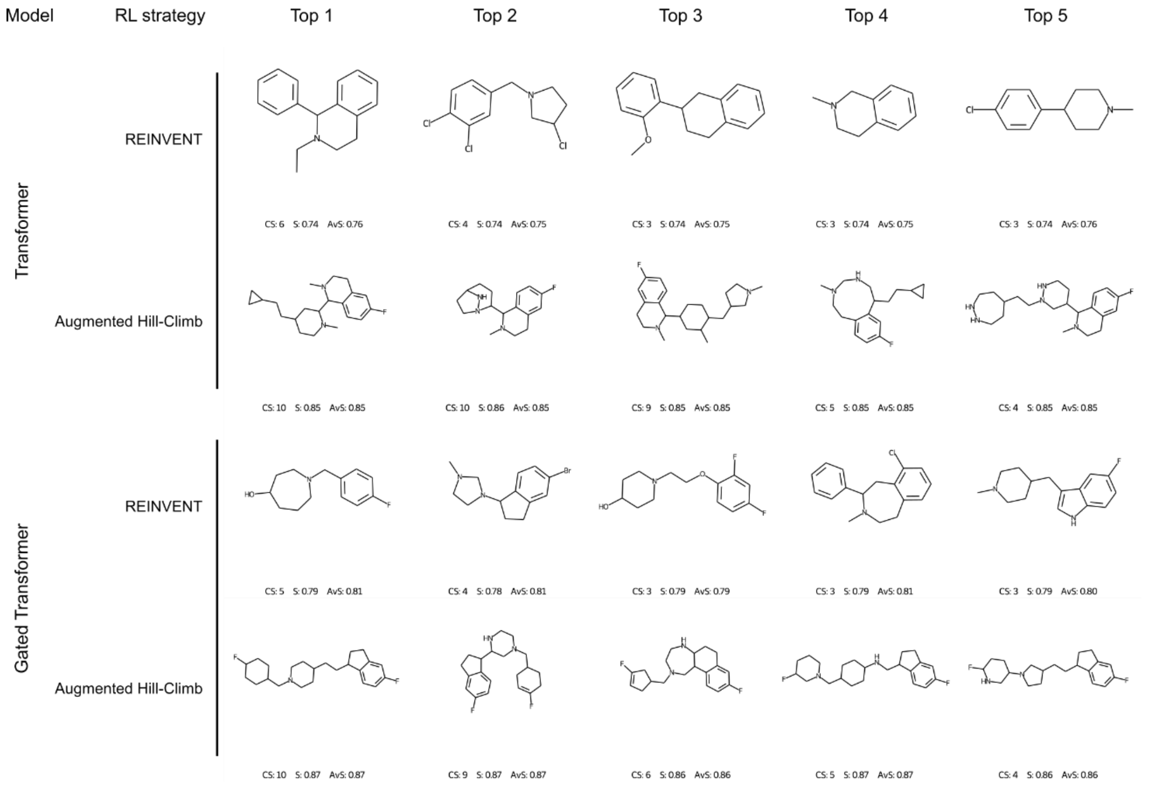

Figure S25: Centroid of the top 5 largest clusters for the top 100 molecules in the DRD2/DRD3 QSAR predicted probability of selective activity task for different transformer models. Cluster size (CS), centroid score (S) and the average cluster score (AvS) is annotated below.
